# Supplementary material for: Stepwise firing mechanism of an extracellular contractile injection system
Source: Nat Commun. 2026 Apr 24;17:5669. doi: 10.1038/s41467-026-72240-y (PMC13314943; doi:10.1038/s41467-026-72240-y)
Supplement: Supplementary file 1 — Supplementary Information [file 41467_2026_72240_MOESM1_ESM.pdf]

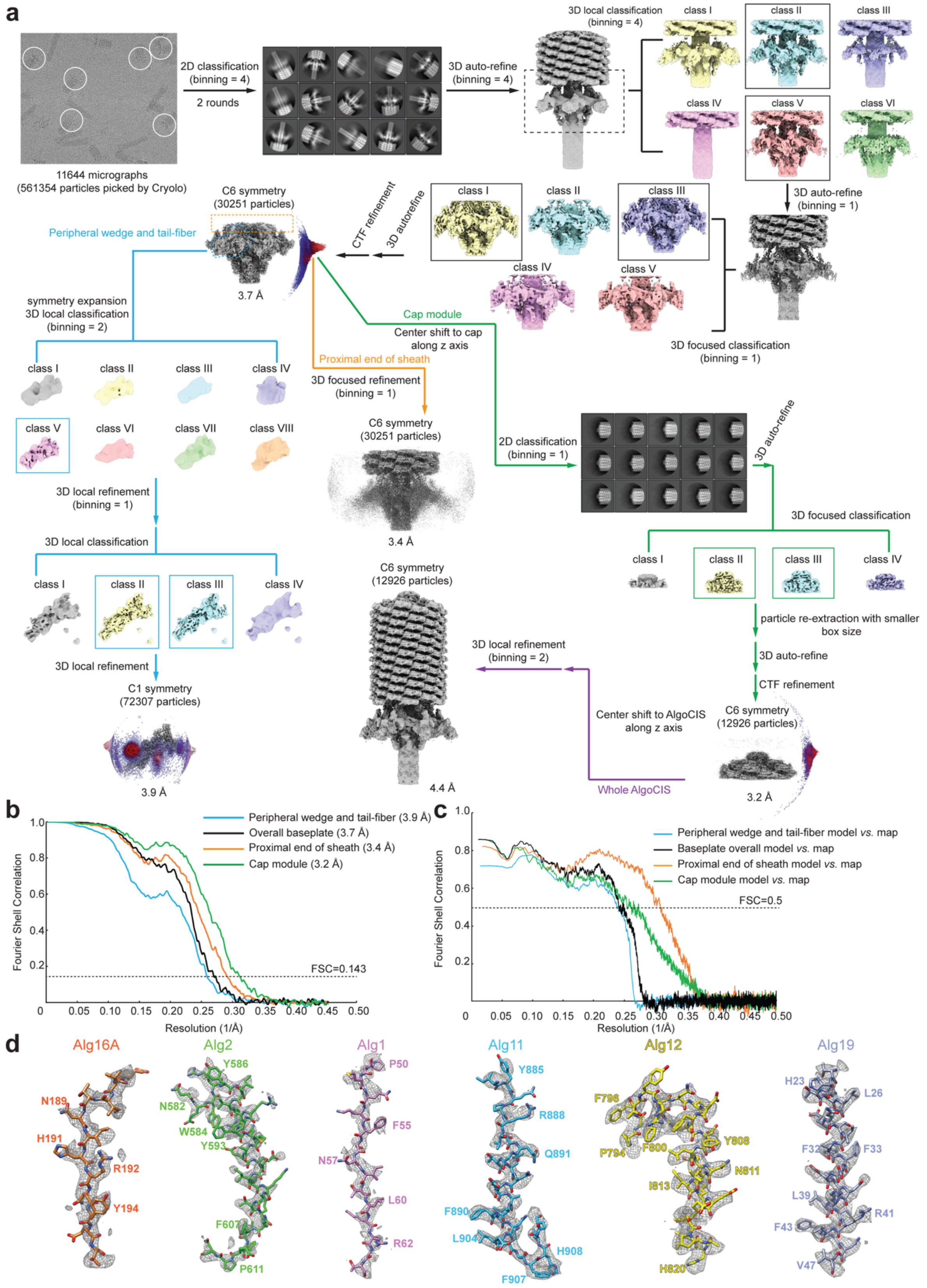
a
11644 micrographs
(561354 particles picked by Cryolo)
2D classification
(binning = 4)
2 rounds
3D auto-refine
(binning = 4)
3D local classification
(binning = 4)
class I
class II
class III
class IV
class V
class VI
3D auto-refine
(binning = 1)
3D focused classification
(binning = 1)
CTF refinement
3D autorefine
C6 symmetry
(30251 particles)
3.7 Å
Peripheral wedge and tail-fiber
symmetry expansion
3D local classification
(binning = 2)
class VII
class VIII
3D local refinement
(binning = 1)
3D local classification
3D local refinement
C1 symmetry
(72307 particles)
3.9 Å
Proximal end of sheath
3D focused refinement
(binning = 1)
C6 symmetry
(30251 particles)
3.4 Å
Cap module
Center shift to cap
along z axis
2D classification
(binning = 1)
3D auto-refine
3D focused classification
particle re-extraction with smaller
box size
3D auto-refine
CTF refinement
C6 symmetry
(12926 particles)
3.2 Å
Center shift to AlgoCIS
along z axis
Whole AlgoCIS
3D local refinement
(binning = 2)
C6 symmetry
(12926 particles)
4.4 Å
b
Fourier Shell Correlation
Resolution (1/Å)
Peripheral wedge and tail-fiber (3.9 Å)
Overall baseplate (3.7 Å)
Proximal end of sheath (3.4 Å)
Cap module (3.2 Å)
FSC=0.143
c
Peripheral wedge and tail-fiber model vs. map
Baseplate overall model vs. map
Proximal end of sheath model vs. map
Cap module model vs. map
FSC=0.5
d
Alg16A
N189
H191
R192
Y194
Alg2
Y586
N582
W584
Y593
F607
P611
Alg1
P50
F55
N57
L60
R62
Alg11
Y885
R888
Q891
F890
L904
H908
F907
Alg12
F796
P794
F800
Y808
N811
I813
H820
Alg19
H23
L26
F32
F33
L39
R41
F43
V47

Supplementary Figure 1 | CryoEM data processing workflow of the post-firing AlgoCIS treated with guanidine-HCl.

a. Flowcharts for the cryoEM reconstruction of different parts of the post-firing AlgoCIS treated with guanidine-HCl. See methods for details.

b-d. Gold-standard Fourier shell correlation (FSC) (**b**) and model *vs.* map FSC (**c**) curves of the cryoEM reconstruction of different parts of the post-firing AlgoCIS treated with guanidine-HCl. The representative densities on different proteins are shown in (**d**).

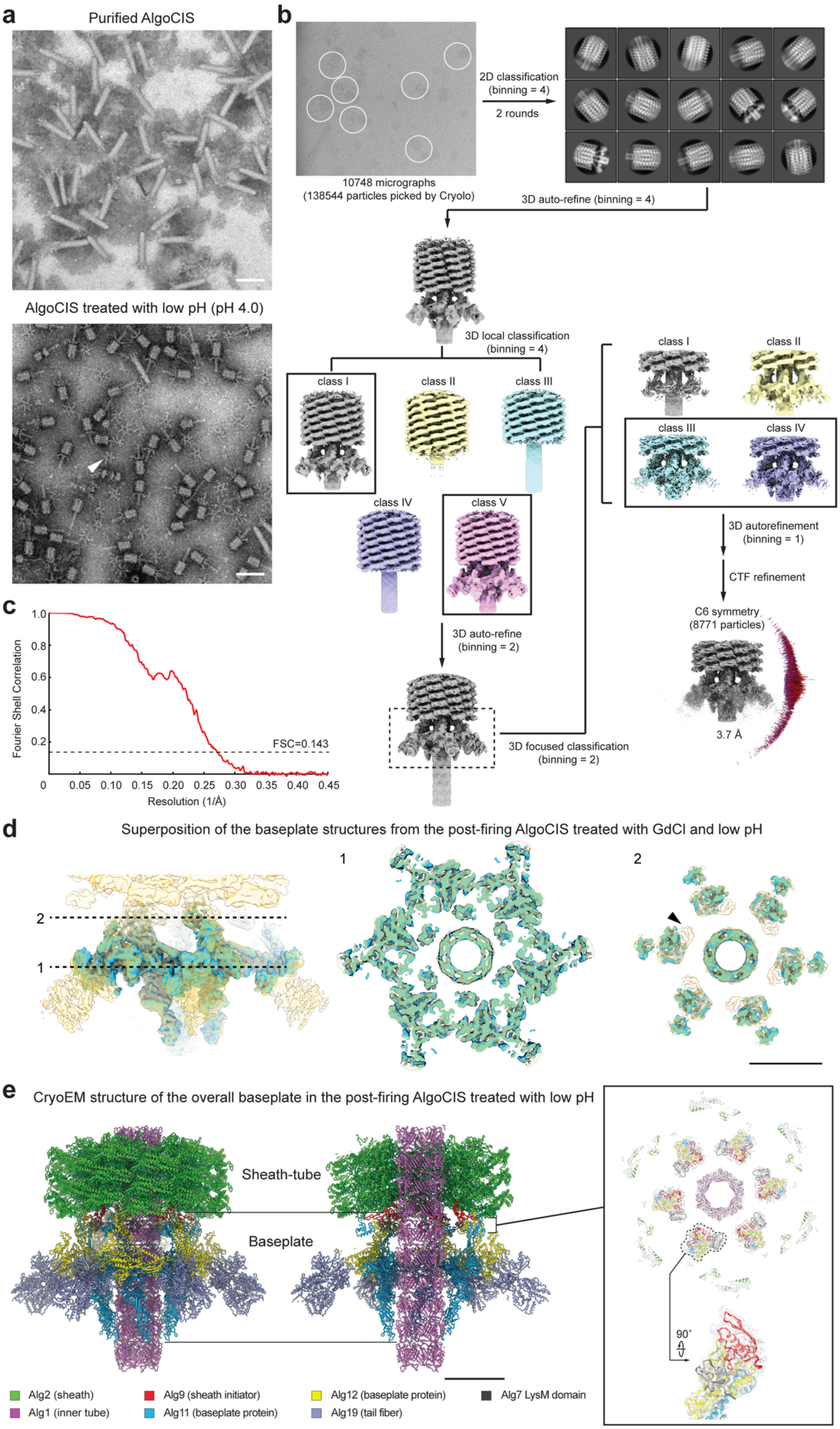

a
Purified AlgoCIS
AlgoCIS treated with low pH (pH 4.0)
b
10748 micrographs
(138544 particles picked by Cryolo)
2D classification
(binning = 4)
2 rounds
3D auto-refine (binning = 4)
3D local classification
(binning = 4)
class I
class II
class III
class IV
class V
3D auto-refine
(binning = 2)
3D focused classification
(binning = 2)
3D autorefinement
(binning = 1)
CTF refinement
C6 symmetry
(8771 particles)
3.7 Å
c
Fourier Shell Correlation
FSC=0.143
Resolution (1/Å)
d
Superposition of the baseplate structures from the post-firing AlgoCIS treated with GdCl and low pH
e
CryoEM structure of the overall baseplate in the post-firing AlgoCIS treated with low pH
Sheath-tube
Baseplate
90°
Alg2 (sheath)
Alg1 (inner tube)
Alg9 (sheath initiator)
Alg11 (baseplate protein)
Alg12 (baseplate protein)
Alg19 (tail fiber)
Alg7 LysM domain

Supplementary Figure 2 | CryoEM data processing workflow of the post-firing AlgoCIS treated with low pH.

a. Negative-stain EM images of purified AlgoCIS with/without low pH treatment, showing that low pH (the value of pH is 4.0) can trigger AlgoCIS firing. Bars: 100 nm. Note that many dissociated baseplate complexes (highlighted by white arrowhead) were observed in the low-pH treated sample.

b. Flowcharts for the cryoEM reconstruction of the baseplate in the post-firing AlgoCIS treated with low pH. See methods for details.

c. Gold-standard FSC curve of the cryoEM reconstruction of the baseplate in the post-firing AlgoCIS treated with low pH.

d. Superposition of the baseplate structures from the post-firing AlgoCIS treated with guanidine-HCl (blue, presented at $\pm 7.6\sigma$) and low pH (orange, shown in transparent, presented at $\pm 3.8\sigma$), showing the overall structural agreement (correlation: 0.98) of the baseplate. The cross-section views at different positions are shown on the right. Both maps were lowpass-filtered to 6 Å. Note that there is one additional density (black arrowhead) observed in the structure from the sample that was treated with low pH, which is formed by LysM domain in Alg7. Bar: 10 nm.

e. Atomic model of the baseplate in the post-firing AlgoCIS treated with low pH (left: side view; middle: central sliced view), showing the almost same structure of the baseplate as the one treated with guanidine-HCl. Cross-section view of the proximal end of the sheath is shown on the top right, where one baseplate wedge is outlined and the side view is shown on the bottom right. Note that LysM domain of Alg7 is found in the post-firing AlgoCIS treated with low pH, but is absent in the model treated with guanidine-HCl. Structural subunits are color-coded as in Fig. 1. Bar: 10 nm.

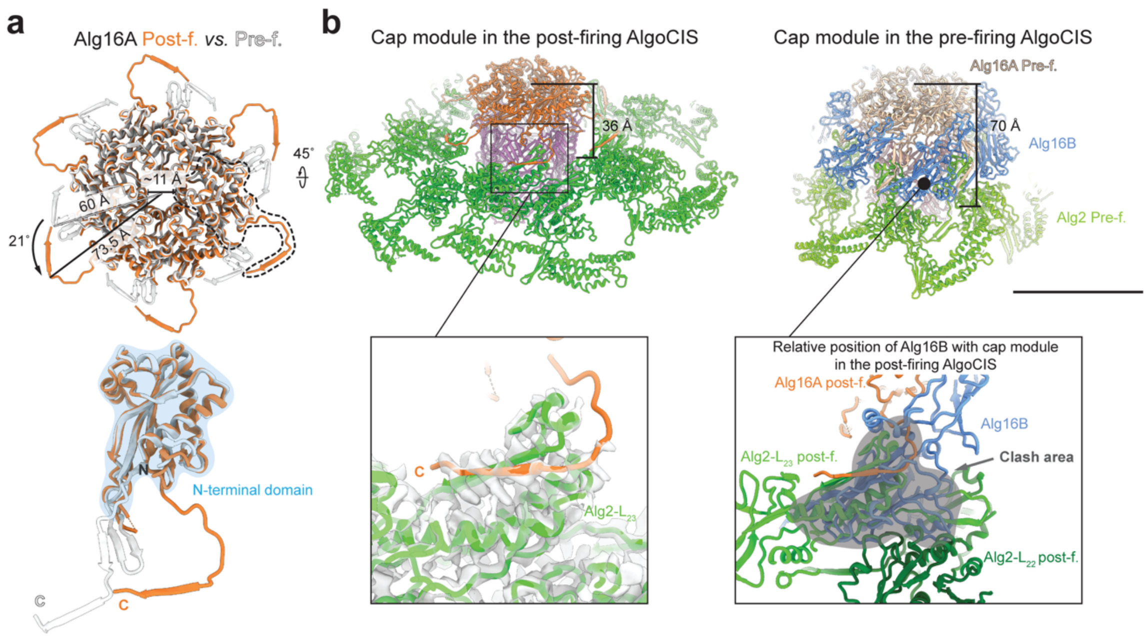

Supplementary Figure 3 | Structural re-arrangement of the cap module in AlgoCIS upon firing.

a. Structural comparison of the cap protein Alg16A in the pre- (white) and post-firing (orange) states, showing that the C-terminus of Alg16A rotates 21˚ upon firing. The inner and outer diameters of six Alg16A proteins were measured. One Alg16A is outlined, and the side view is shown on the bottom, with N-terminal domain shadowed in cyan and the N- and C-termini labeled.

b. Structural comparison of the cap module of in the post- (left) and the pre-firing (right) AlgoCIS, showing that the cap adaptor (Alg16B: blue) is absent in the post-firing AlgoCIS. The distance between the cap and the proximal sheath layer (Alg2-L_{23}) decreases upon firing (contracted: 36 Å *vs.* extended: 70 Å). The contact sites between the cap and the distal sheath layer are zoomed-in and shown on the bottom. Note that severe clashes (shadowed in grey) are observed between the cap adaptor (in the pre-firing state) and the distal sheath layer (in the post-firing state) when superpositioned based on the N-terminal domain of the cap, suggesting that the cap adaptor is extruded upon firing. Structural subunits are color-coded as Fig. 1, the cap protein (Alg16A) and the distal sheath layer in the pre-firing state is colored brown and light green. The second distal sheath layer (Alg2-L_{22}) in the post-firing state is colored dark green. The EM map is shown transparent. Bar: 10 nm.

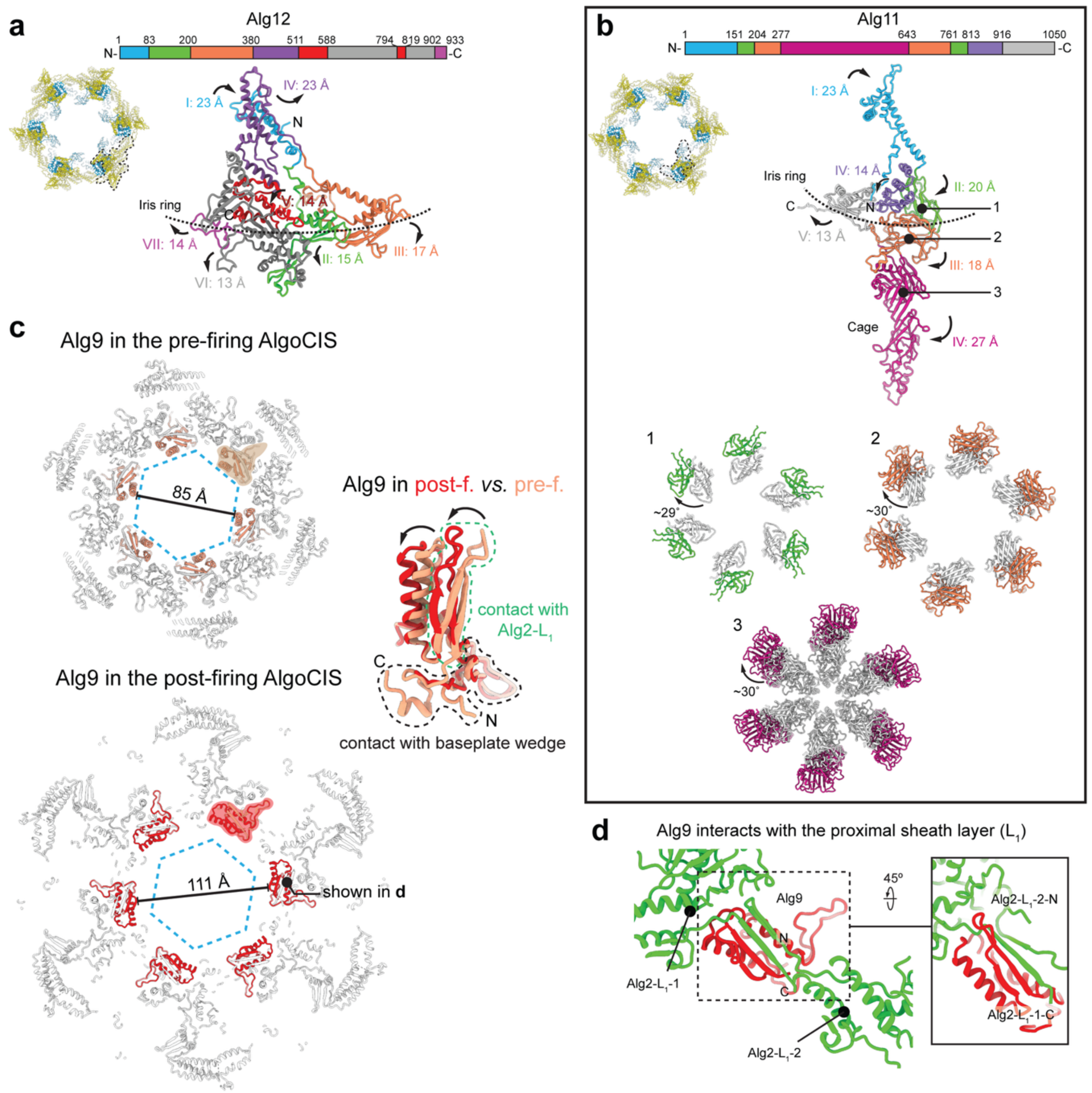

Supplementary Figure 4 | Structural re-arrangement of the baseplate in AlgoCIS upon firing.

a. Individual domains of Alg12 have different degrees of outward movement upon firing. Left: Cross-section view of the iris (color-coded as in Fig. 1), one Alg12 protein is outlined. Right: Domain organization of Alg12 (top) and the structure of the Alg12 protein in the post-firing state (bottom). Domains are color-coded, the position of the iris is represented by dashed line. Movements of individual domains are indicated by arrows, where the values were measured based on the shifts of the centers of each domain in the pre- and post-firing states.

b. Individual domains of Alg11 have different degrees of outward movement upon firing. Top: Cross-section view of the iris (shown as panel **a**), where one Alg11 protein is outlined. Middle: Domain organization of Alg11 (top) and the structure of the Alg11 protein in the post-firing state

(bottom). Style is shown same as in (**a**). The movements of individual domains are indicated by arrows and are measured. Bottom: Cross-section views on different domains of Alg11 showing that the domain II (1), domain III (2), and domain IV (3, cage) have both rotation (~30˚, indicated by arrows) and outward expansion upon firing. The Alg11 proteins in the pre-firing state are colored white.

c. Structural comparison of the sheath initiator protein (Alg9) in the pre- (top-left, coral) and post-firing (bottom-left, red) states, showing that Alg9 has outward movement upon firing. The diameters of Alg9 rings are labeled, the positions of the inner tube are presented by the dashed hexagon (blue). One Alg9 protein in the pre- and post-firing states are shadowed and the structural comparison is shown on the right. The structural re-arrangements are indicated by arrows, the regions that interact with the proximal sheath layer or the baseplate Alg11/12 heterodimer are outlined by green or black dashed line, respectively.

d. Alg9 interacts with two adjacent sheath proteins via hand-shake interactions in the post-firing state. Structural subunits are color-coded as in Fig. 1.

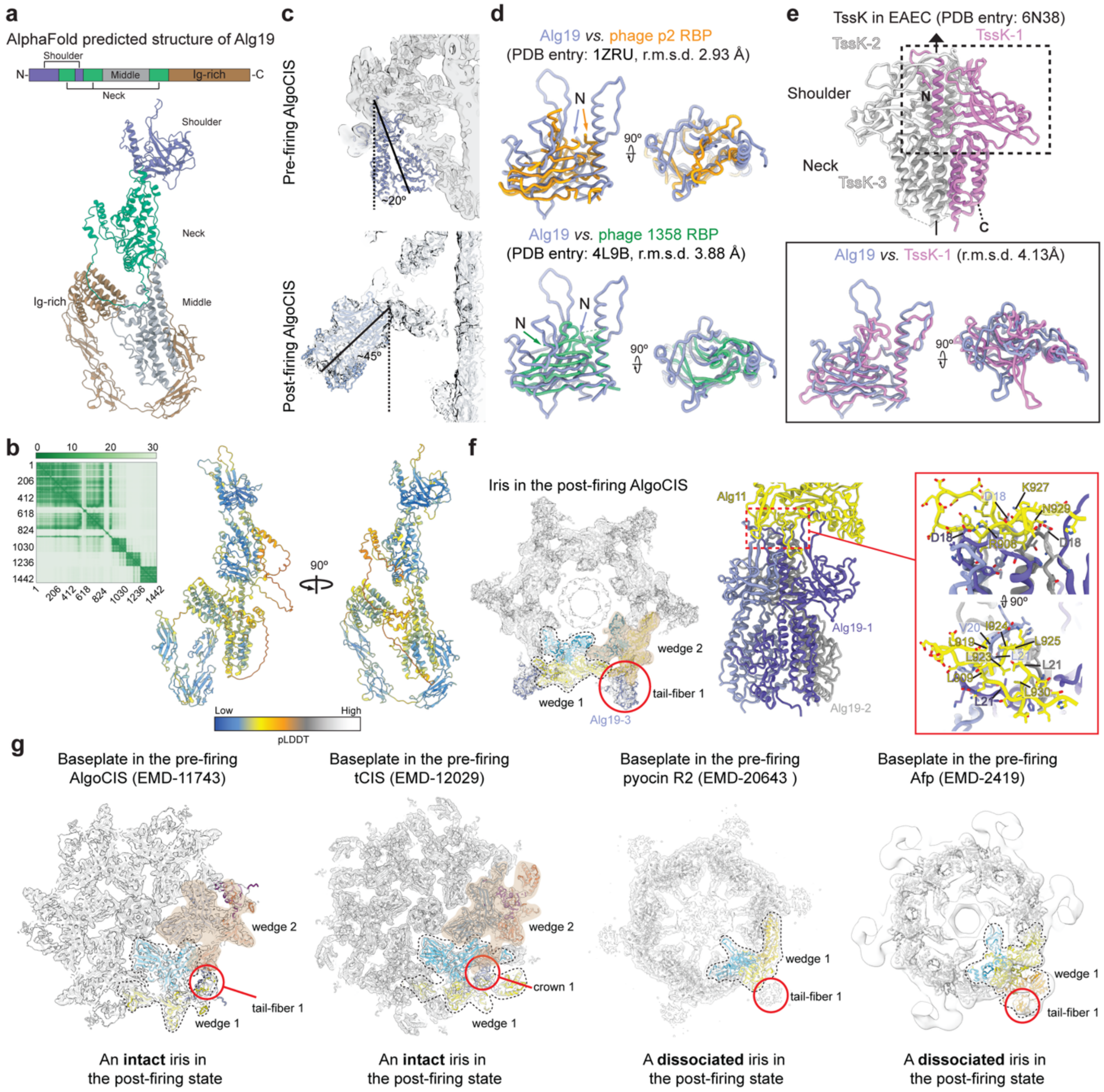

Supplementary Figure 5 | Tail-fibers bind to the baseplate.

a-b. Alg19 is predicted to have four domains. Domain organization and Alphafold predicted structure of Alg19 is shown in panel **a**, the domains are color-coded. The detail Alphafold analysis (left: PAE plot; right: pLDDT distribution) was shown in panel **b**.

c. Tail-fiber undergoes substantial conformational changes upon firing. The structures of tail-fiber (gray) in the pre- (left)/post-firing (right) AlgoCIS are shown and the angles against particle axis (vertical axis) are measured. The pre-firing AlgoCIS map is lowpass-filtered to 8 Å.

d. The shoulder domain of Alg19 has a similar structure as some phage receptor binding proteins (RBPs) (top: lactococcal phage p2 RBP, 1ZRU, orange; bottom: lactococcal phage 1358 RBP, 4L9B, green). The overall RMSD values are indicated.

e. The shoulder domain of Alg19 has a similar structure as the shoulder part of the T6SS TssK in enteroaggregative *Escherichia coli*. Trimeric TssK (6N38) is shown on the top, one protomer is colored pink. The shoulder domain of TssK is highlighted by dashed box, while the structural superposition with Alg19 is shown on the bottom. The overall RMSD value is indicated.

f. One tail-fiber connects two adjacent baseplate wedges. Left: cross-section view of the baseplate in the post-firing AlgoCIS. One baseplate wedge is outlined and subunits (wedge 1) are color-coded as in Fig. 1. The adjacent wedge (wedge 2) is shadowed in brown and subunits are colored (Alg11: coral; Alg12: dark blue; tail-fiber: magenta). The tail-fiber binding site is highlighted by circle. Right: side view of one tail-fiber binding to the baseplate. The binding sites are boxed and the zoom-in is shown on the right. The residues participating in contacts are presented with side chain and are labeled.

g. The tail-fibers have different binding profiles to the baseplate among CISs. Shown are cross-section views of the baseplate from different CISs in the pre-firing state. The structures are shown in the same style as panel (**f**). Note that one tail-fiber binds to one baseplate wedge in pyocin R2 and Afp, which has a dissociated iris upon firing, while one tail-fiber/crown connects two baseplate wedges in AlgoCIS/tCIS that keeps an intact iris upon firing.

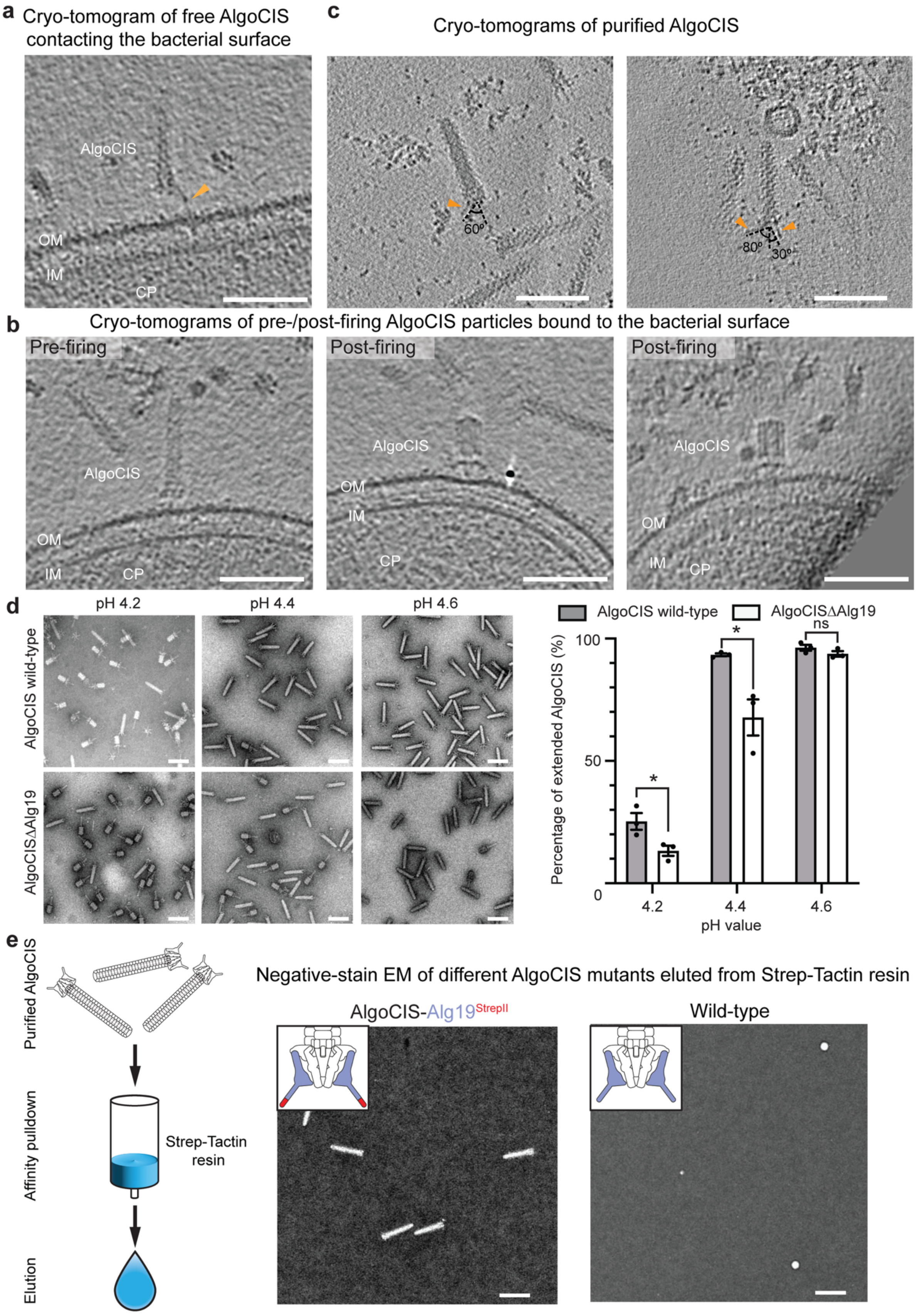
a
Cryo-tomogram of free AlgoCIS contacting the bacterial surface
AlgoCIS
OM
IM
CP
c
Cryo-tomograms of purified AlgoCIS
60º
80º
30º
b
Cryo-tomograms of pre-/post-firing AlgoCIS particles bound to the bacterial surface
Pre-firing
AlgoCIS
OM
IM
CP
Post-firing
AlgoCIS
OM
IM
CP
Post-firing
AlgoCIS
OM
IM
CP
d
pH 4.2
pH 4.4
pH 4.6
AlgoCIS wild-type
AlgoCISΔAlg19
AlgoCIS wild-type
AlgoCISΔAlg19
Percentage of extended AlgoCIS (%)
100
50
0
*
*
ns
4.2
4.4
4.6
pH value
e
Purified AlgoCIS
Affinity pulldown
Strep-Tactin resin
Elution
Negative-stain EM of different AlgoCIS mutants eluted from Strep-Tactin resin
AlgoCIS-Alg19StrepII
Wild-type

Supplementary Figure 6 | Tail-fibers mediate the AlgoCIS attachment to bacterial surface.

a-b. Additional examples in cryo-tomograms of co-incubation of AlgoCIS wild-type with *E. pacifica*. A pre-firing AlgoCIS particle contacts the bacterial surface via its flexible tail-fiber (**a**, orange arrowhead). A pre-firing AlgoCIS perpendicularly attaches to the bacterial surface (**b**, left panel). AlgoCIS fired on the bacterial surface (**b**, middle and right panels). Shown are projections of 18 nm thick slices. Bars: 100 nm. OM: outer membrane; IM: inner membrane; CP: cytoplasm.

c. Cryo-tomograms showing that the C-terminal part of the tail-fiber is highly flexible in non-attached AlgoCIS. Shown are projections of 10.8 nm. The tail-fibers are indicated with orange arrowheads, and the angles are measured. Bars: 100 nm.

d. AlgoCIS is more sensitive to low pH treatment when knocking out the tail-fibers. Shown are representative negative-stain EM images of wild-type and the tail-fiber knockout mutant (AlgoCISΔAlg19) upon treatments with different pH values. Quantification is shown on the right. Note that some AlgoCISΔAlg19 contract in the buffer with pH value of 4.4, but almost all particles of wild-type remain in the pre-firing state. Plotted values show the mean ± SD from three independent experiments. Statistical significance was calculated using T-test. Bars: 100 nm. The experiment was repeated three times. The raw counting results were provided in Source Data.

e. Schematic (left) and negative-stain EM images (right) showing that the purified AlgoCIS fused with C-terminal StrepII-tag (AlgoCIS-Alg19^{StrepII}) remains in the pre-firing state after StrepII-tag pulldown, indicating that the binding of the tail-fiber to a receptor is insufficient to trigger the firing. The wild-type is regarded as negative control. Bars: 100 nm.

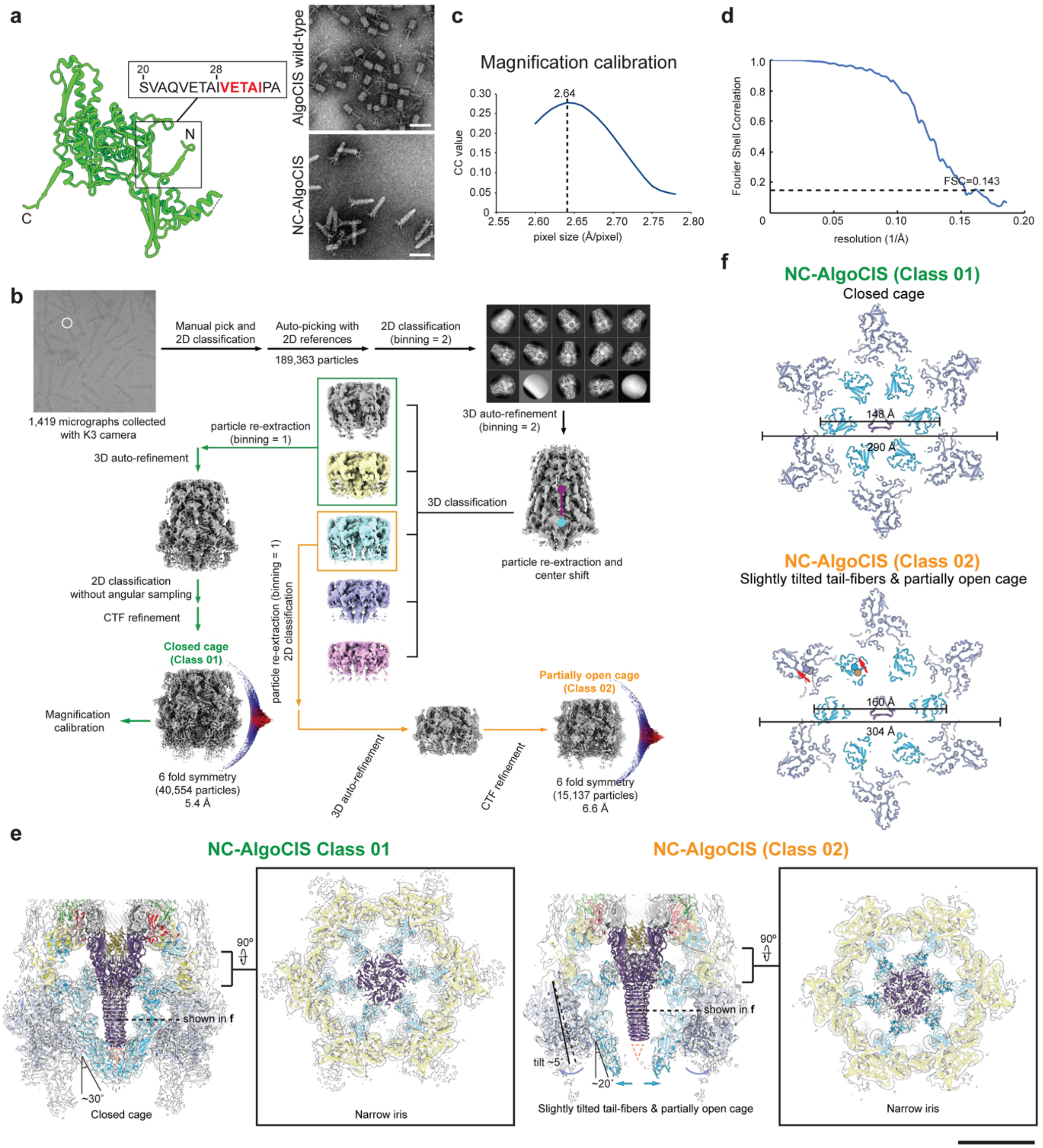

Supplementary Figure 7 | CryoEM analyses of an AlgoCIS intermediate state.

a. Re-engineering of the non-contractile AlgoCIS (NC-AlgoCIS) mutant by introducing five additional amino acid residues (red) into the N-terminal part of sheath protein Alg2. Representative negative-stain EM images are shown on the right. Note that NC-AlgoCIS cannot contract upon low pH (pH value of 4.0) treatment, while all wild-type particles fire in the same condition. Bars: 100 nm.

b. Flowcharts for the cryoEM reconstruction of the non-contractile AlgoCIS mutant (NC-AlgoCIS) treated with low pH. See methods for details. Note that two classes of NC-AlgoCIS were determined from the dataset: one is in the pre-firing state and has a closed cage (Class 01); the other is in the pre-firing state but has slightly tilted tail-fibers and a partially opened cage (Class 02).

c. Plot of magnification calibration on the dataset of NC-AlgoCIS treated with low pH. See methods for details.

d. Gold-standard FSC curve of the cryoEM reconstruction of the baseplate with slightly tilted tail-fibers and a partially opened cage in NC-AlgoCIS (Class 02) treated with low pH.

e-f. NC-AlgoCIS with slightly tilted tail-fibers has a partially opened cage upon low pH treatment. Shown are the two classes of NC-AlgoCIS treated with low pH. The central sliced views of the overall baseplate structure (left) and the cross-section view of the iris (right) are shown in panel **e**. Structural re-arrangements on the cage and tail-fibers are indicated by arrows (blue: cage; dark gray: tail-fibers). The angles between the cage and the perpendicular axis were measured. The outward tilted angle is labeled, where the axis of the tail-fibers with/without outward tilting is presented with a solid or dashed line, respectively. The cross-section view on the tail-fibers is shown in panel **f**. The outer-diameters of the cage and the tail-fibers were measured. The shifts of the centers of the cage (Class 01: orange; Class 02: blue) and the tail-fibers (Class 01: brown; Class 02: dark grey) are indicated by red arrows. Bar: 10 nm.

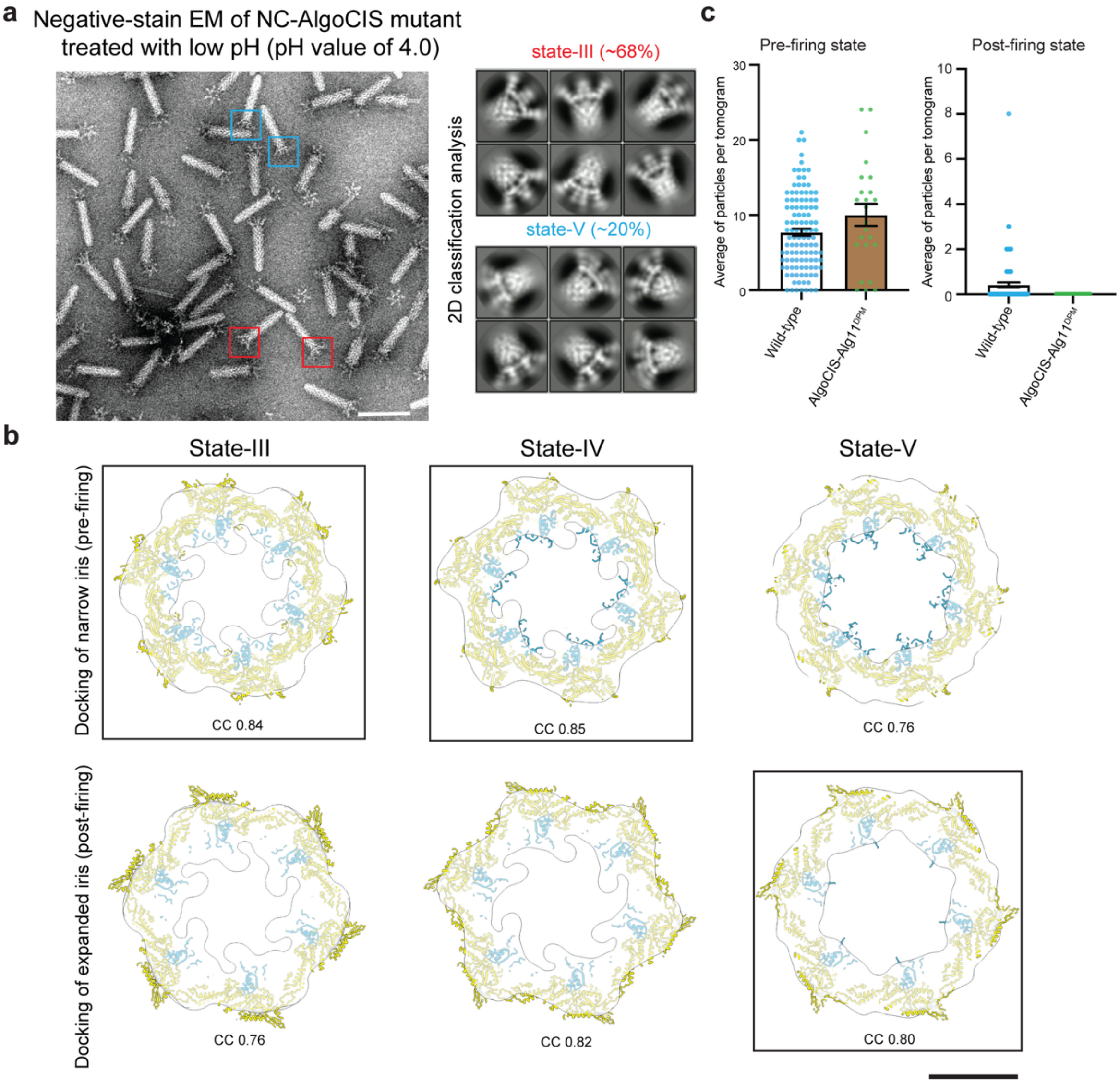

Supplementary Figure 8 | Different intermediates are observed in NC-AlgoCIS treated with low pH.

a. Two different intermediate structures were observed when NC-AlgoCIS was treated with low pH and imaged by negative-stain EM. Left: one representative negative-stain EM image of NC-AlgoCIS upon low pH treatment, where two intermediates are highlighted by colored boxes (state-III: red; state-V: blue). Right: 2D classes of two intermediates (state-III: top; state-V: bottom), the percentages of each state are indicated. Bar: 100 nm.

b. Structural fittings of the pre-(top)/post-firing (bottom) iris into reconstructed maps of intermediate structures, showing that the iris of state-III and state-IV are in the pre-firing state, and the iris of state-V is in the post-firing state. Correlation coefficient (CC) values are stated. Iris structures with best CC value are boxed. See methods for details. Bar: 10 nm.

c. Quantification of the pre- (left)/post-firing (right) AlgoCIS mutants on the surface of *E. pacifica* in cryo-tomograms (n = 112 in wild-type; n = 24 in AlgoCIS-Alg11^{DPM}). Data are shown as mean ± SEM. Note that no firing events are observed in *E. pacifica* co-incubated with AlgoCIS-Alg11^{DPM}. The dataset of wild-type is the same as shown in Fig. 3c. The counting of wild-type was from three batches of dataset, while AlgoCIS-Alg11^{DPM} was from one batch of dataset. The raw counting results were provided in Source Data.

Supplementary Table 1 | CryoEM data statistical analysis

	Contracted AlgoCIS treated with GmCl				Contracted AlgoCIS treated with low pH	NC-AlgoCIS treated with low pH	
	Cap (EMD-66213, 9WT1)	Baseplate iris structure (EMD-66211, 9WSZ)	Proximal end of sheath (EMD-66214)	Peripheral wedge and tail-fiber (EMD-66212, 9WT0)	Baseplate overall (EMD-69920)	Baseplate in the extended state (EMD-69921)	Baseplate with partially open cage (EMD-69922)
**Data collection and processing**							
Nominal magnification	81,000					33,000	
Voltage (kV)	300					200	
Electron exposure ($e^-/Å^2$)	60 (K3 camera)						
Defocus range (µm)	1.0-3.0						
Pixel size (Å/pixel)	1.1					2.64 (calibrated)	
Symmetry imposed	C6	C6	C6	C1	C6	C6	C6
Final particles (No.)	12,926	30,251	30,251	72,307	8,771	40,554	15,137
Map resolution	3.2	3.7	3.4	3.9	3.7	5.4	6.6
FSC threshold	0.143						
**Refinement**							
Map sharpening B factor ($Å^2$)	-64	-54	-82	-58	-52	-98	0
Model composition							
Non-hydrogen atoms	80766	92154		18756	-	-	-
Protein residues	11088	11406		2329	-	-	-
Chains	30	12		4	-	-	-
R.M.S deviations							
Bond length (Å)	0.015	0.017		0.017	-	-	-
Bond angles (°)	1.403	1.554		1.619	-	-	-
Validation							
MolProbity score	1.38	1.46		1.31	-	-	-
Clashscore	5.81	5.17		2.81		-	-
Rotamer outlier (%)	0.08	0.06		0.05	-	-	-
Ramachandran plot							
Favored (%)	97.73	96.92		96.42	-	-	-
Allowed (%)	2.05	3.08		3.58	-	-	-
Outlier (%)	0.22	0.00		0.00	-	-	-
Masked CC	0.67	0.72	0.82	0.67	-	-	-

Supplementary Table 2 | List of strains, plasmids and primers in this study

Strains

Strain ID	Name	From
CFE 060	*Algoriphagus machipongonensis* PR1	DSM 24695 (https://www.dsmz.de/collection/catalogue/details/culture/DSM-24695)
CFE 130	*A. machipongonesis* alg2-N5 (non-contractile)	(This study)
CFE 116	*A. machipongonesis* Δalg19	(This study)
CFE 163	*A. machipongonesis* alg11-C268A	(This study)
CFE 159	*A. machipongonesis* alg11-C1000A	(This study)
CFE 161	*A. machipongonesis* alg11-C268A,C1000A	(This study)
CFE 261	*A. machipongonesis* alg19-CT_Strep	(This study)
pCFE 004	*E.coli* SM10 (λpir)	From Shikuma Lab

Plasmids

Plasmid ID:	Plasmid Name:	Function:	From:
pCFE 078	pCHIP3	Create mutants in A. machipongonesis PR1	Xu, 2022
pCFE 106	pCHIP3_alg2-N5	Create the non-contractile AlgoCIS	(This Study)
pCFE 103	pCHIP3_ Δalg19	Create the Tail fiber (alg19) deletion	(This Study)
pCFE 116	pCHIP3_alg11-C268A	Change the 268 cystine to an alanine	(This Study)
pCFE 117	pCHIP3_alg11-C1000A	Change the 1000 cystine to an alanine	(This Study)
pCFE 118	pCHIP3_alg11-C268A,C1000A	To disrupt the 268-1000 disulfide bond	(This Study)
pCFE 154	pCHIP3_alg19-CT-Strep	Add a Strep tag to the Cterm of alg19	(This Study)
pCFE 121	pCHIP3_alg11Δcage	To delete the cage in AlgoCIS	(This Study)

Primers

Primer ID:	Name	Sequence	Function	From
primCE 263	pCHIP3_SeqF1	TTCTGTTGCATGGGCATAAA	To sequence cloning plasmids	Xu, 2022
primCE 265	pCHIP3_SeqR2	CGTGAATTCAAAGGGAGAGC	To sequence cloning plasmids	Xu, 2022
primCE 259	alg2_dA	GTTAAAAAGGATCGATCCTCTAGATGGGGGACTTTAGGAGGAAG	Create the non-contractile AlgoCIS	(This Study)
primCE 356	alg2-N5_B1	GGGATTGCGGTTTCTACGATTGCGGTTTCTACTTGTGCTACAGAGGGGGGA	Create the non-contractile AlgoCIS	(This Study)
primCE 357	alg2-N5_C1	TCCCCCCTCTGTAGCACAAGTAGAAACCGCAATCGTAGAAACCGCAATCCC	Create the non-contractile AlgoCIS	(This Study)
primCE 262	alg2_dD	CGTGAATTCAAAGGGAGAGCTCACACATTGAGCAACGAGCTG	Create the non-contractile AlgoCIS	(This Study)
primCE 358	alg19_dA	GTTAAAAAGGATCGATCCTCTAGAAAATTGGGTTTATCGCTGGA	Create the Tail fiber (alg19) deletion	(This Study)
primCE 359	alg19_dB	CTTTACTTGTTCCAAGAAAGACGGTAAAGATTAAAGAGTTC	Create the Tail fiber (alg19) deletion	(This Study)
primCE 360	alg19_dC	GAACTCTTTAATCTTTACCGTCTTTCTTGGAACAAGTAAAG	Create the Tail fiber (alg19) deletion	(This Study)
primCE 361	alg19_dD	CGTGAATTCAAAGGGAGAGCTCAATCCTTGGCAAATTGATGC	Create the Tail fiber (alg19) deletion	(This Study)
primCE 362	alg19_SeqF1	AAGAGGAAAGGCTTTATAGCATCA	To sequence the tail fiber contructs	(This Study)
primCE 363	alg19_SeqR1	TGGAATTCGACCATCACCTT	To sequence the tail fiber contructs	(This Study)
primCE 364	alg19_SeqF2	TCGAGGTTCATGGAAAGTCC	To sequence the tail fiber contructs	(This Study)
primCE 365	alg19_SeqR2	ATTCGGCTTACAATCGCATC	To sequence the tail fiber contructs	(This Study)
primCE 282	alg11_dA	GTTAAAAAGGATCGATCCTCTAGATTGAAACCCCAACAGGAAAC	To disrupt the 268-1000 disulfide bond	Xu, 2022
primCE 412	alg11-C268A_B1	CTGGGAGTACGGTAGAAGCATTCGCAATTTTGGTATAGCCAAAAG	To disrupt the 268-1000 disulfide bond	(This Study)
primCE 413	alg11-C268A_C1	CTTTTGGCTATACCAAAATTGCGAATGCTTCTACCGTACTCCCAG	To disrupt the 268-1000 disulfide bond	(This Study)
primCE 414	alg11-C1000A_B1	GTTTCAATAAAAATGGGGTTCGCAGGAGATGAACCAGCTTGAC	To disrupt the 268-1000 disulfide bond	(This Study)
primCE 415	alg11-C1000A_C1	GTCAAGCTGGTTCATCTCCTGCGAACCCCATTTTTATTGAAAC	To disrupt the 268-1000 disulfide bond	(This Study)
primCE 285	alg11_dD	CGTGAATTCAAAGGGAGAGCTCCGATCCCATCATTTTCATCA	To disrupt the 268-1000 disulfide bond	Xu, 2022
primCE 286	alg11_SeqF1	GGGGATTTCCACCAGAATTT	To sequence the alg11 point mutations	(This Study)
primCE 287	alg11_SeqR1	TCCTTTGACCCCTCCTTCTT	To sequence the alg11 point mutations	(This Study)
primCE 611	alg19-Strep-Linker_B1	TTATTTTTCGAACTGCGGGTGGCTCCAACCAGAA CCACCTACCATTAAAGTCAATTGA	Add a Strep tag to the Cterm of alg19	(This Study)
primCE 612	alg19-Strep-Linker_C1	GGTGGTTCTGGTTGGAGCCACCCGCAGTTCGAA AAATAATTTCTTGGAACAAGTAAAG	Add a Strep tag to the Cterm of alg19	(This Study)
primCE 432	alg11Δcage_B1	CTCGCCCCCATGGCAATAGCTACCGCTGCCGCTA CCCGCTTTAGGAAGTGCTGGGA	To delete the baseplate cage in AlgoCIS	(This Study)
primCE 433	alg11Δcage_C1	TCCCAGCACTTCCTAAAGCGGGTAGCGGCAGCGG TAGCTATTGCCATGGGGGCGAG	To delete the baseplate cage in AlgoCIS	(This Study)
