# Supplementary material for: Stepwise firing mechanism of an extracellular contractile injection system
Source: Nat Commun. 2026 Apr 24;17:5669. doi: 10.1038/s41467-026-72240-y (PMC13314943; doi:10.1038/s41467-026-72240-y)
Supplement: Supplementary file 2 — Reporting Summary [file 41467_2026_72240_MOESM2_ESM.pdf]

nature portfolio

Corresponding author(s): Martin Pilhofer, Jingwei Xu

Last updated by author(s): 2026/03/31

Reporting Summary

Nature Portfolio wishes to improve the reproducibility of the work that we publish. This form provides structure for consistency and transparency in reporting. For further information on Nature Portfolio policies, see our Editorial Policies and the Editorial Policy Checklist.

Statistics

For all statistical analyses, confirm that the following items are present in the figure legend, table legend, main text, or Methods section.

n/a	Confirmed	
☐	☒	The exact sample size (*n*) for each experimental group/condition, given as a discrete number and unit of measurement
☐	☒	A statement on whether measurements were taken from distinct samples or whether the same sample was measured repeatedly
☒	☐	The statistical test(s) used AND whether they are one- or two-sided<br>*Only common tests should be described solely by name; describe more complex techniques in the Methods section.*
☒	☐	A description of all covariates tested
☒	☐	A description of any assumptions or corrections, such as tests of normality and adjustment for multiple comparisons
☐	☒	A full description of the statistical parameters including central tendency (e.g. means) or other basic estimates (e.g. regression coefficient) AND variation (e.g. standard deviation) or associated estimates of uncertainty (e.g. confidence intervals)
☒	☐	For null hypothesis testing, the test statistic (e.g. *F*, *t*, *r*) with confidence intervals, effect sizes, degrees of freedom and *P* value noted<br>*Give P values as exact values whenever suitable.*
☒	☐	For Bayesian analysis, information on the choice of priors and Markov chain Monte Carlo settings
☒	☐	For hierarchical and complex designs, identification of the appropriate level for tests and full reporting of outcomes
☒	☐	Estimates of effect sizes (e.g. Cohen's *d*, Pearson's *r*), indicating how they were calculated

Our web collection on statistics for biologists contains articles on many of the points above.

Software and code

Policy information about availability of computer code

Data collection	SerialEM-3.8.6 was used for cryoET data collection. For cryoEM data, SerialEM-3.8.6 was used to collect the dataset of the post-firing AlgoCIS treated with GdCl, EPU (3.10.0.8733) was used to collect the dataset of the post-firing AlgoCIS treated with low pH. All negative staining data were collected manually.
Data analysis	MotionCor2_1.1.0, Gctf-v1.06_sm_30, Relion-3.1, UCSF Chimera-1.13, UCSF ChimeraX-1.1, COOT-0.8.9.1, PHENIX-1.13-2998, ImageJ-1.53f51 (Fiji), IMOD-4.11.0, Rosetta-2018.09.60072, diffmap_120330, Prism v10, isonet, Dynamo-1.1.401

For manuscripts utilizing custom algorithms or software that are central to the research but not yet described in published literature, software must be made available to editors and reviewers. We strongly encourage code deposition in a community repository (e.g. GitHub). See the Nature Portfolio guidelines for submitting code & software for further information.

Data

Policy information about availability of data

All manuscripts must include a data availability statement. This statement should provide the following information, where applicable:

- Accession codes, unique identifiers, or web links for publicly available datasets
- A description of any restrictions on data availability
- For clinical datasets or third party data, please ensure that the statement adheres to our policy

The cryoEM density maps and corresponding atomic models have been deposited in the EMDB and PDB, respectively. The accession numbers are listed as following:EMD-66211 and 9WSZ (the baseplate iris structure in the post-firing state of AlgoCIS), EMD-66212 and 9WT0 (the tail fiber bound to the baseplate wedge

in the post-firing state of AlgoCIS), EMD-66213 and 9WT1 (the cap module in the post-firing state of AlgoCIS), EMD-66214 (the proximal part of sheath-tube module connected to the baseplate in the post-firing state of AlgoCIS), EMD-69920 (the baseplate iris structure in the post-firing state of AlgoCIS upon low-pH treatment), EMD-69921 (the baseplate iris structure in the pre-firing state of non-contractile sheath AlgoCIS mutant upon low-pH treatment), EMD-69922 (the baseplate iris structure with partially open cage of non-contractile sheath AlgoCIS mutant upon low-pH treatment), EMD-69923 (sub-tomogram average of pre-firing AlgoCIS perpendicularly bound to bacterial surface), EMD-69924 (sub-tomogram average of post-firing AlgoCIS perpendicularly bound to bacterial surface), EMD-69925 (cryo-tomogram of pre-/post-firing AlgoCIS perpendicularly bound to bacterial surface).
The previously published structures used in this work includes phage p2 receptor binding protein (1ZRU), phage 1358 receptor binding protein (4L9B), TssK in enteroaggregative Escherichia coli (6N38), cryoEM density map of the baseplate in the pre-firing AlgoCIS (EMD-11743), cryoEM density map of the baseplate in the pre-firing tCIS (EMD-12029), cryoEM density map of the baseplate in the pre-firing pyocin R2 (EMD-20643), cryoEM density map of the baseplate in the pre-firing Afp (EMD-2419).

Research involving human participants, their data, or biological material

Policy information about studies with human participants or human data. See also policy information about sex, gender (identity/presentation), and sexual orientation and race, ethnicity and racism.

Reporting on sex and gender	This study does not involve human participants. It therefore has no report on sex and gender.
Reporting on race, ethnicity, or other socially relevant groupings	This study does not involve human participants. It therefore has no report on race, ethnicity, or other socially relevant groupings.
Population characteristics	This study does not involve human participants. It therefore has no report on population characteristics.
Recruitment	This study does not involve human participants. It therefore has no report on recruitment.
Ethics oversight	This study does not involve human participants. It therefore has no report on ethics oversight.

Note that full information on the approval of the study protocol must also be provided in the manuscript.

Field-specific reporting

Please select the one below that is the best fit for your research. If you are not sure, read the appropriate sections before making your selection.

☒ Life sciences ☐ Behavioural & social sciences ☐ Ecological, evolutionary & environmental sciences

For a reference copy of the document with all sections, see nature.com/documents/nr-reporting-summary-flat.pdf

Life sciences study design

All studies must disclose on these points even when the disclosure is negative.

Sample size	Sample size was stated in the main text, methods as well as relevant figure legends. For example, we analyzed 112 tomograms of wild-type AlgoCIS incubated with E. pacifica, while 24 tomograms of AlgoCISΔAlg19 were used (mentioned in Fig. 3b). For cryoEM dataset, we collected 11644 micrographs of AlgoCIS treated GdCl, 10748 micrographs of AlgoCIS treated with low-pH, 1419 micrographs of NC-AlgoCIS treated with low-pH, as mentioned in Method part.
Data exclusions	No data was excluded from the analyses preformed.
Replication	All data was confirmed with proper biological replicates (please see details in related figure legends) to ensure reproducibility of the assays shown in the study. Biological replicates of both the eukaryotic as well as the bacteria or sample preparations were used were the cultures were grown from frozen stock to readout independently of the related replicates.
Randomization	Assays in the study did not need to be randomized. Eukaryotic cells were grown up in appropriate growth media before being quantified to allow for the same number of cells to be placed into each well for the assays to keep the experiments consistent.
Blinding	Blinding was not relevant to our study.

Reporting for specific materials, systems and methods

We require information from authors about some types of materials, experimental systems and methods used in many studies. Here, indicate whether each material, system or method listed is relevant to your study. If you are not sure if a list item applies to your research, read the appropriate section before selecting a response.

Materials & experimental systems

n/a	Involved in the study
☒	☐ Antibodies
☒	☐ Eukaryotic cell lines
☒	☐ Palaeontology and archaeology
☒	☐ Animals and other organisms
☒	☐ Clinical data
☒	☐ Dual use research of concern
☒	☐ Plants

Methods

n/a	Involved in the study
☒	☐ ChIP-seq
☒	☐ Flow cytometry
☒	☐ MRI-based neuroimaging

Plants

Seed stocks	This research is not relevant to plants.
Novel plant genotypes	This research is not relevant to plants.
Authentication	This research is not relevant to plants.
