# Supplementary material for: Stepwise firing mechanism of an extracellular contractile injection system
Source: Nat Commun. 2026 Apr 24;17:5669. doi: 10.1038/s41467-026-72240-y (PMC13314943; doi:10.1038/s41467-026-72240-y)
Supplement: Supplementary file 3 — Transparent Peer Review file [file 41467_2026_72240_MOESM3_ESM.pdf]

nature portfolio

Peer Review File

Stepwise firing mechanism of an extracellular contractile injection system

Corresponding Author: Professor Martin Pilhofer

This file contains all reviewer reports in order by version, followed by all author rebuttals in order by version.

Version 0:

Reviewer comments:

Reviewer #1

(Remarks to the Author)
The manuscript by Xu et al. entitled “Stepwise firing mechanism of an extracellular contractile injection system” describes the pre- and post-firing states as well as four intermediate firing states of the contractile injection system (CIS) from the marine bacterium Algoriphagus machipongonensis (AlgoCIS) studied by single particle cryo-EM, cryo-ET, and structure-guided engineering. The structures allow for a first detailed description of signal propagation leading to and including firing. Structures of the pre- and post-firing states, solved by both single particle cryo-EM and subtomogram averaging of cryo-ET data, made it possible to identify key interactions, residues and a disulfide bond. These findings allowed for the design of new mutants to mimic four intermediate states and their structure determination by single particle cryo-EM.

Interestingly, the order of information in the manuscript follows experimental order, which works well due to the logic and required background for experiments. Key findings include the critical nature of Alg9 for initiating sheath contraction. Furthermore, firing forces the cap adaptor Alg16B proteins to dissociate and Alg16B is thus not present in the post-firing structure. Combined with previous data of a knockout mutant, the authors conclude that in the pre-firing state, Alg16B stabilizes and “locks” AlgoCIS. The authors determined the importance of tail-fiber binding to targets, where they found evidence of individual tail-fibers interacting with bacterial surfaces via cryo-ET and sub-tomogram averaging. In fact, this revealed additional densities compared to the purified samples, likely to be tail-fiber protein Alg19, further supporting knockout mutant data on the key role of Alg19 in cell surface binding. The structures of several states allowed the authors to identify different conformations of the iris and the cage, and to propose that the signal that triggers firing involves the cage and the iris. They could specifically identify a disulfide bond that was essential for firing. A double-mutant allowed them to understand additional intermediates. Including the pre- and post-firing wild-type structures, a total of 6 structures allowed the authors to propose a model for a stepwise firing mechanism.

The manuscript provides substantial contributions that will be of interest to a wide audience: In addition to (1) the impressive and extensive information on the firing mechanism, the studies allowed the authors to (2) identify notable similarities and differences compared to phage and other CISs. As the authors also emphasize, (3) the overall approach can be applied to understand the mechanism of similar as well as distantly related systems. Moreover, (4) the detailed understanding of a number of AlgoCIS proteins and their interactions will allow for engineering to target specific cells by delivery of cargo proteins as AlgoCIS is an eCIS, which is of high interest for biomedical engineering.

Minor comments:

p. 4, l. 74-77: I recommend briefly explaining why specifically GdCl and low pH were used. The wider audience of the journal will require some brief background for the strategy.

p. 4, l. 75: Please include details on the resolution here in addition to the information already provided in the extended data table. The resolution provides critical context for the atomic model and the conclusions on the mechanism.

p. 5, l. 120: Please clarify why the prediction by Alphafold is mentioned here.

p. 8, l. 198-199: Please clarify this sentence: What is meant by the signal traveling through the interaction between the cage and the iris?

Figures:

Fig. 5c: “partically” should read “partially”

Fig. 5c: The magenta hexagons are too small to easily identify.

Extended Data Fig. 5d: It would be helpful to move or copy this figure to Fig. 2 as it contains critical information.

Reviewer #2

(Remarks to the Author)
The work by Xu et al used high-resolution cryo-electron microscopy approach with structure-guided engineering to reveal stepwise mechanisms of the firing and contraction of a bacterial contractile injection system, AlgoCIS. The manuscript provided a high resolution of contracted structure of AlgoCISs triggered by GdCl or low pH, in particular the structure of baseplate iris, with comparison to its extended state. Importantly, they also analyzed the post-firing structure of purified AlgoCISs binding to bacterial cell surface, revealing the function of tail fiber in cell surface attachment and stabilization of pre-firing state. Next, using structure-guided engineering, authors have used different mutants to capture several intermediate states of contraction, thus subsequently revealed a stepwise firing event. This study significantly expands and deepens our current understanding of contraction of CISs and will inspire and guide future investigations into further mechanical insights of other CISs. The manuscript is very well prepared and clearly written, and the structures revealed are of high resolution. The methodology is sound with good data analyses. With such solid data, the work is convincing. Below, I have provided a few points that need to be clarified, or potentially worth addressing or discussing to strengthen the study.
1. My most major point is about the potential difference revealed when the contraction is triggered by different ‘triggers’: GdCl, low-pH or cell surface binding. For example, although the contracted structures triggered by GdCl and low-pH are quite similar, there was still difference such as additional density observed in extended data figure 3d (line 406-408). While authors have stated that it was the lysM domain of Alg7 in the figure legend, there was no further discussion in the text and speculation of why. Furthermore, it will be worth discussing how biologically relevant such conditions (GdCl or low-pH) are, how mechanically such conditions initiate the contraction (via baseplate or sheath subunits? As it does not look like via tail fiber as cell surface binding does?), and how they differ from surface receptor binding triggered firing? Clearer presentation of the differences and more discussion on these will be appreciated.
2. It appeared that there were only 5% of the properly surface-binding AlgoCISs that were in post-firing state (Fig3c and line 150-152). How about free floating particle? In addition, between line 230-231, it was stated that ‘some particles were loosely bound to the surface’, could you provide the percentage number? In both cases, it raises the question of whether this bacterium is the actual target of AlgoCISs. It would be nice if author elaborates more on this (for example, what were other potential target cells? Can one get a hint from the cargo/effector proteins?). Also, was there anything noticeable on the morphology of E. pacifica cells that have contracted AlgoCISs attached? Furthermore, as authors have the purified AlgoCISs with Alg19 fused with StrepII, it is perhaps possible to perform immunoprecipitation assay of AlgoCISs harboring StrepII-Alg19 co-incubating with E. pacifica, to identify the receptors, if it is within the feasibility and scope of this work.
3. Line 94-95 and extended figure 4c: as there was only one replicate of SDS gel followed by mass spec analysis of one faint band, it may need proper label-free proteomic quantification with triplicate to conclude that there was less Alg16B post firing. Such quantification will also include the quantity of most other Alg proteins, which should remain the same and serve as controls. In addition, from the gel, there was a band missing in post-firing samples around size of 25 kDa, which could be interesting to know what it was.
4. State III (fully tilted tail fibers and closed cage): while there were quite a number of particles at state III (68%), however, when this state could be during the proposed stepwise process was not clear in Fig 5 or discussed enough. Especially compared to state II, where tail fibers were slightly tilted but cage has been already partially open. If state II was the stage before state III, then does it mean that cage opens a bit then closes, and only when the tail fiber is fully tilted, then it reopens fully (entering state IV)?
5. Figure 5d: the state of ‘fully tilted tail fibers, fully open cage, narrow iris’ appeared to have close cage by the illustration above the text (if I understood correctly); also, what were the wavy lines indicating? Thus, it would be good to include the legend for color/shape representation of different proteins or structure parts in this figure.

Other minor points:
1. Line 67 –‘suggesting a firing mechanism that may be distinct from pyocin R2’: more explanation why authors hypothesize so will be appreciated for broader audience, since the checksum mechanism for pyocin R2 was also too briefly mentioned without more detailed comparison with the stepwise mechanism presented here.
2. There was sometimes inconsistency in reference style. For example, line 138 (and several places throughout the text like line 233, 254 etc..) had style like ‘pyocin R2 (ref.15)’, which was different from the rest of the text.
3. Line 204: while AlgoCIS-Alg1DPM could not bind to bacterial cell surface, how about the non-contractile sheath mutant NC-AlgoCIS? If they bind, were there also different intermediate states observed?
4. Line 350 – ‘The position of the PAAR-like protein Alg10 is represented by dashed triangles’: It should be clarified whether Alg10 was seen here by these structural analyses, or it was assumed that they were positioned there but not resolved within the analyses.
5. Extended data figure 3a: noticeably, there were a large number of starfish shape-like arrangements of thin tubes, could authors indicate/speculate what they were within the figure?

Reviewer #3

(Remarks to the Author)
This paper tackles a central question in the field of type VI secretion systems and associated contractile injection systems: how is contraction/injection triggered and carried out? The authors choose Algoriphagus machipongonensis's AlgoCIS as a model system and start be determining the structure of the contracted form for comparison to a previously-published uncontracted form. They then investigate the role of the tail fibres, including an experiment to investigate whether tail fibre alone is sufficient to trigger contraction (it isn't). A series of serendipitous discoveries and rational insights subsequently lead the authors to capture a series of snapshots of the firing process of AlgoCIS: they design a non-contractile sheath mutant to stall the firing process and determined an intermediate structure using cryo-EM after firing was triggered. Negative staining of the same sample revealed two additional structural states. Finally, they generated a mutant lacking a disulphide bond in Alg11, which connects the cage and iris regions of the system, to investigate how the firing signal is transmitted through different CIS components. This led to the identification of another new structural state. By arranging these structures in sequence, the authors proposed a stepwise firing mechanism for the CIS.

The manuscript is overall very well-written, although perhaps a little lacking in details in places, likely an attempt to fit to word count. The science is good and I have no concerns, although some written commentary on choices would benefit the reader. Figures are overall very communicative although would benefit from additional information as described below.

Major points

As mentioned repeatedly in my Minor points, I had some difficulty following ther narrative of the paper due to choices of what to put in Extended Data. In many cases I found that information essential to understanding the manuscript was in Extended, necessitating that flip back and forth to find it. I appreciate that length restrictions mean that some important data goes in Extended Data, but the core of a paper should be stand-alone and not absolutely require reference to essential data in Extended. Rearrangement of Figures is needed to render this manuscript more easily accessible (the writing, on the other hand, is overall very good). In a similar vein in the text, many assumptions are not explained sufficiently for a non-expert reader understand the rationale behind choices, and a number of critical details are buried in Extended Data or Methods. I describe some, but not all, of these issues below.

I also have concerns regarding the rationale for the interpretation of the stepwise order of firing. Given that the non-contractile sheath mutant stalls at state II, it seems that sheath contraction occurs prior to cage opening and tail fiber tilting, and is necessary for these conformational changes. Therefore, rather than reflecting the physiological firing pathway of the CIS in vivo, the transitions observed under negative staining may represent an alternative series of conformational changes occurring in the absence of sheath contraction. It would greatly clarify the proposed mechanism if the authors discussed the role of the sheath protein (Alg2) in mediating structural rearrangements during firing. Similarly it isn't clear why the authors choose place so-called "stage III" after "stage II", given that the cage is more open in "stage II", which is arguably more important for injection of the tube that tail fibre pivoting.

Minor points

Introduction, line 44: the description of the "checksum" mechanism here fails to relate the name "checksum" to the described mechanism. For an audience who are not experts in the field, explanation of why the mechanism is referred to as "checksum" would be helpful.

Introduction, line 47: this paragraph uses the phrase "mode of action" to refer to the broader context in which they act. I'd argue a better phrase might be something like, "the context in which these CISs work differs". To me, "Mode of action" strongly evokes their firing mechanism (the gap in knowledge that you seek to address).

Results, line 73: Would be nice to have the nominal resolution stated instead of having to have readers search for this information.

Figure 1 (and Extended Data Figure 1): is the operon structure relevant? Consider removing? At the very least it should be a separate panel. I personally did not find that this contributed to the story, yet critical information was relegated to Extended Data figures (more on which below).

Results, line 81 / Figure 1: This is about comparison to the previously-published uncontracted state (indeed, the figure title is "AlgoCIS undergoes significant structural re-organization upon firing."), but it feels awkward to flip between Extended Data Figure 1 and Figure 1 to make the comparisons between published and unpublished, and Figure 1 cannot stand alone. I'd strongly recommend combining the two figures into a larger figure, and include the electron density map for the unconstricted state. Extended Data should extend a figure, but not be an essential part of the figure that is relegated to the end of the paper: Extended Data Figure 1 is an essential part of Figure 1 and therefore deserves to be merged into the main figure.

Line 111 and throughout: As a non expert, I struggled to understand what structures correspond to the “cage” - please highlight in Figure 1. An interpretative cartoon would be invaluable here (like the last column of Fig. 4), and include definition of the iris (which is currently defined in Fig. 2).

Figure 2: my comments are the same here as Figure 1. Figure 2 currently does not stand alone, and needs Extended Data Figure 4 to be merged into it. A detail panel of Alg19 pivoting might also be helpful here instead of referring back to a part of Fig. 1?

Line 163: I would be interested to hear your speculations on why any AlgoCIS can bind without Alg19?

Line 170: Nice experiment!

Line 182: I’m not sure if “generating” insights is quite the right phrase? “Attain” insights, or “generate data”?

Figure 4: Figure 4 feels more like an interpretative figure than a data figure. Again, this would benefit from incorporating some of Extended Data Figure 9. Perhaps the more interpretative aspects could be moved to Figure 5, which is already semi-redundant with Figure 4, and Figure 4 fleshed-out with more actual data?

Line 193: I agree that this is exciting, and appreciate that some peculiarity of the system necessitates negative stain work. I would appreciate a little more interpretative context from the authors, though: why do you think negative stain was required for these insights? Do you have concerns over whether these may be artefacts? If not, why not? I don’t view these as issues, but think the paper would be improved with this commentary.

Line 201: I’d have appreciated some context. Why did you think this disulphide bond is relevant to firing?

Line 203: Define “DPM”.

Fig. 5C: How do the tail fibres first bind to the outer leaflet of the outer membrane – which is NOT fluid – and subsequently splay out to enable the cage to come to close proximity with the membrane? Once bound the tail fibres would be locked immobile by LPS.

Version 1:

Reviewer comments:

Reviewer #2

(Remarks to the Author)
The work by Xu et al used high-resolution cryo-EM with structure-guided engineering to reveal stepwise mechanisms of the firing and contraction of a bacterial contractile injection system, AlgoCIS. The contraction of CISs was previously poorly understood, however, this manuscript has revealed a novel mechanism, which is distinct from the previous 'checksum' mechanism proposed for pyocin R2.

With the new revision, authors have successfully addressed all my review comments and confusions I had (I really appreciate their clear explanation in addressing comments, not only in the manuscript, but also in the letter). With the revision, I do not have further major comments (with only one minor comment listed below). In general, with such extensive solid data on the high resolution structural analyses, the work is very impressive and convincing. This study significantly expands and deepens our current understanding on the firing of CISs and will guide future investigations into further mechanical insights of the contraction of other CISs. Moreover, I also believe that it will be of interest to a wide audience. For example, this cleverly designed structure-guided engineering approach could inspire future efforts to “lock” other dynamic nanomachines at specific stages, enabling the resolution of stepwise mechanisms that are otherwise difficult to capture due to their too dynamic, transient or subtle conformational changes.

minor comment: since the author decides to remove the original ED fig. 4c of the SDS PAGE and Mass spec analysis on the pull-down assays, perhaps majority of the method section on this part can be removed (line 654-672), unless it is relevant to other data.

Reviewer #3

(Remarks to the Author)
I am happy with the author's revisions. Very nice!

Open Access This Peer Review File is licensed under a Creative Commons Attribution 4.0 International License, which permits use, sharing, adaptation, distribution and reproduction in any medium or format, as long as you give appropriate credit to the original author(s) and the source, provide a link to the Creative Commons license, and indicate if changes were made.
In cases where reviewers are anonymous, credit should be given to 'Anonymous Referee' and the source.
The images or other third party material in this Peer Review File are included in the article's Creative Commons license, unless indicated otherwise in a credit line to the material. If material is not included in the article's Creative Commons license and your intended use is not permitted by statutory regulation or exceeds the permitted use, you will need to obtain permission directly from the copyright holder.
To view a copy of this license, visit https://creativecommons.org/licenses/by/4.0/

Reviewers' comments in black
Authors' responses in blue

Reviewer #1 (Remarks to the Author)
The manuscript by Xu et al. entitled "Stepwise firing mechanism of an extracellular contractile injection system" describes the pre- and post-firing states as well as four intermediate firing states of the contractile injection system (CIS) from the marine bacterium Algoriphagus machipongonensis (AlgoCIS) studied by single particle cryo-EM, cryo-ET, and structure-guided engineering. The structures allow for a first detailed description of signal propagation leading to and including firing. Structures of the pre- and post-firing states, solved by both single particle cryo-EM and subtomogram averaging of cryo-ET data, made it possible to identify key interactions, residues and a disulfide bond. These findings allowed for the design of new mutants to mimic four intermediate states and their structure determination by single particle cryo-EM.

Interestingly, the order of information in the manuscript follows experimental order, which works well due to the logic and required background for experiments. Key findings include the critical nature of Alg9 for initiating sheath contraction. Furthermore, firing forces the cap adaptor Alg16B proteins to dissociate and Alg16B is thus not present in the post-firing structure. Combined with previous data of a knockout mutant, the authors conclude that in the pre-firing state, Alg16B stabilizes and "locks" AlgoCIS. The authors determined the importance of tail-fiber binding to targets, where they found evidence of individual tail-fibers interacting with bacterial surfaces via cryo-ET and sub-tomogram averaging. In fact, this revealed additional densities compared to the purified samples, likely to be tail-fiber protein Alg19, further supporting knockout mutant data on the key role of Alg19 in cell surface binding. The structures of several states allowed the authors to identify different conformations of the iris and the cage, and to propose that the signal that triggers firing involves the cage and the iris. They could specifically identify a disulfide bond that was essential for firing. A double-mutant allowed them to understand additional intermediates. Including the pre- and post-firing wild-type structures, a total of 6 structures allowed the authors to propose a model for a stepwise firing mechanism.

The manuscript provides substantial contributions that will be of interest to a wide audience: In addition to (1) the impressive and extensive information on the firing mechanism, the studies allowed the authors to (2) identify notable similarities and differences compared to phage and other CISs. As the authors also emphasize, (3) the overall approach can be applied to understand the mechanism of similar as well as distantly related systems. Moreover, (4) the detailed understanding of a number of AlgoCIS proteins and their interactions will allow for engineering to target specific cells by delivery of cargo proteins as AlgoCIS is an eCIS, which is of high interest for biomedical engineering.

We share enthusiasm of reviewer and have revised the manuscript accordingly. Thank you for the suggestions.

Minor comments:
p. 4, l. 74-77: I recommend briefly explaining why specifically GdCl and low pH were used. The wider audience of the journal will require some brief background for the strategy.

We added sentences to explain potential functions of GdCl (line 75-76) and low-pH treatment (line 82).

p. 4, l. 75: Please include details on the resolution here in addition to the information already provided in the extended data table. The resolution provides critical context for the atomic model and the conclusions on the mechanism.

We included information on resolution in different parts of post-firing AlgoCIS (line 77-79).

p. 5, l. 120: Please clarify why the prediction by Alphafold is mentioned here.

Since the protein size of Alg19 is relatively big (1443 residues) and its structure has not been determined so far, we performed Alphafold prediction to analyze its domain organization. We explained our rationale in the revised version (line 125-126).

p. 8, l. 198-199: Please clarify this sentence: What is meant by the signal traveling through the interaction between the cage and the iris?

Since the state-III has a narrow iris but tilted tail-fibers, it is likely that tilting of tail-fibers only is not sufficient to trigger the iris expansion. The structural difference between state-III (narrow iris, closed cage, tilted tail-fibers) and state-V (expanded iris, open cage, and tilted tail-fibers) indicated that the cage possibly opens prior to the iris expansion. We therefore speculated that the conformational change of the cage will generate a signal that propagates to the iris via contacts between them. The iris will then expand and further initiate sheath contraction.

We have clarified accordingly in the revised manuscript (line 207-213).

Figures:
Fig. 5c: "partically" should read "partially"

We have revised accordingly.

Fig. 5c: The magenta hexagons are too small to easily identify.

We have made hexagons much bigger in the revised version.

Extended Data Fig. 5d: It would be helpful to move or copy this figure to Fig. 2 as it contains critical information.

Since sheath initiator protein Alg9 has the same interaction with sheath protein Alg2 in both of the pre-/post-firing AlgoCIS and it is conserved in other CISs, we do not think that it is necessary to move ED. Fig 5d to Fig. 2.

Reviewer #2 (Remarks to the Author)
The work by Xu et al used high-resolution cryo-electron microscopy approach with structure-guided engineering to reveal stepwise mechanisms of the firing and contraction of a bacterial contractile injection system, AlgoCIS. The manuscript provided a high resolution of contracted structure of AlgoCISs triggered by GdCl or low pH, in particular the structure of baseplate iris, with comparison to its extended state. Importantly, they also analyzed the post-firing structure of purified AlgoCISs binding to bacterial cell surface, revealing the function of tail fiber in cell surface attachment and stabilization of pre-firing state. Next, using structure-guided engineering, authors

have used different mutants to capture several intermediate states of contraction, thus subsequently revealed a stepwise firing event. This study significantly expands and deepens our current understanding of contraction of CISs and will inspire and guide future investigations into further mechanical insights of other CISs. The manuscript is very well prepared and clearly written, and the structures revealed are of high resolution. The methodology is sound with good data analyses. With such solid data, the work is convincing. Below, I have provided a few points that need to be clarified, or potentially worth addressing or discussing to strengthen the study.

We appreciate the positive feedback from the reviewer.

1. My most major point is about the potential difference revealed when the contraction is triggered by different 'triggers': GdCl, low-pH or cell surface binding. For example, although the contracted structures triggered by GdCl and low-pH are quite similar, there was still difference such as additional density observed in extended data figure 3d (line 406-408). While authors have stated that it was the lysM domain of Alg7 in the figure legend, there was no further discussion in the text and speculation of why. Furthermore, it will be worth discussing how biologically relevant such conditions (GdCl or low-pH) are, how mechanically such conditions initiate the contraction (via baseplate or sheath subunits? As it does not look like via tail fiber as cell surface binding does?), and how they differ from surface receptor binding triggered firing? Clearer presentation of the differences and more discussion on these will be appreciated.

Please see our response to Reviewer 1. We explained potential functions of GdCl and low-pH treatment. Since sheath is highly stable and we frequently observed the dissociated baseplate complex ("starfish-like" complex in ED Fig. 2a), we speculated that different chemical treatments here mainly disrupt contacts between individual baseplate subunits (as mentioned in PMID: 32350467), resulting in sheath contraction. Moreover, we described the presence of the LysM domain and speculated about the possible reason in the revised text (line 84-86).

The resolution of post-firing AlgoCIS on the bacterial surface is fairly low (~60 Å). We therefore cannot retrieve more detailed structural differences when docking the cryoEM structure of post-firing AlgoCIS, except that we observed more densities in the distal end of tail-fibers. We described this observation in the revised version (line 169-171).

2. It appeared that there were only 5% of the properly surface-binding AlgoCISs that were in post-firing state (Fig3c and line 150-152). How about free floating particle? In addition, between line 230-231, it was stated that 'some particles were loosely bound to the surface', could you provide the percentage number? In both cases, it raises the question of whether this bacterium is the actual target of AlgoCISs. It would be nice if author elaborates more on this (for example, what were other potential target cells? Can one get a hint from the cargo/effector proteins?). Also, was there anything noticeable on the morphology of E. pacifica cells that have contracted AlgoCISs attached? Furthermore, as authors have the purified AlgoCISs with Alg19 fused with StrepII, it is perhaps possible to perform immunoprecipitation assay of AlgoCISs harboring StrepII-Alg19 co-incubating with E. pacifica, to identify the receptors, if it is within the feasibility and scope of this work.

For free floating AlgoCIS particles, we first analyzed one cryo-tomogram and found that the percentage of post-firing AlgoCIS was extremely low (0.6%, 5 out of 772 particles, Figure R1). We then performed quantification analysis on the full dataset. Indeed, the number of free-floating

post-firing AlgoCIS is very low (183 particles in 114 tomograms), whereas there are hundreds of pre-firing particles in one tomogram. These data show that free-floating particles are stable after purification, which is similar for other eCISs (e.g. Afp and PVC).

For the percentage of loosely bound AlgoCIS, we found that it is not easy to confidently identify them in cryo-tomograms, because the C-terminal part of tail-fibers are sometimes too flexible to be visualized. We therefore cannot provide the accurate percentage of loosely bound AlgoCIS.

To explore the targets of AlgoCIS, we previously showed that the cargo protein Cgo1 has toxic effect when heterogeneously expressed in *E. coli* (PMID: 35165385). We hypothesized that AlgoCIS might target bacteria and then performed screenings of purified AlgoCIS against diverse species. We however did not find any promising candidates based on the phenotype of cell death.

Surprisingly, we here visualized that AlgoCIS can fire and penetrate bacterial outer membrane. Based on slightly higher percentage of post-firing AlgoCIS on bacterial surface (~5%) compared to free-floating (~0.6%), it is possible that *E. pacifica* is one of targets of AlgoCIS. Interestingly, the bacteria with post-firing AlgoCIS did not have significant morphological difference in cryo-tomograms. AlgoCIS might perform other effects against *E. pacifica* rather than killing the host. Moreover, we also imaged the co-incubation of AlgoCIS and other bacteria (*E. coli*, data not shown), but we did not observe clear contacts.

To identify the receptor of AlgoCIS, we appreciate the ideas from reviewer, however, these experiments were not feasible in the timeline for the revision of this manuscript.

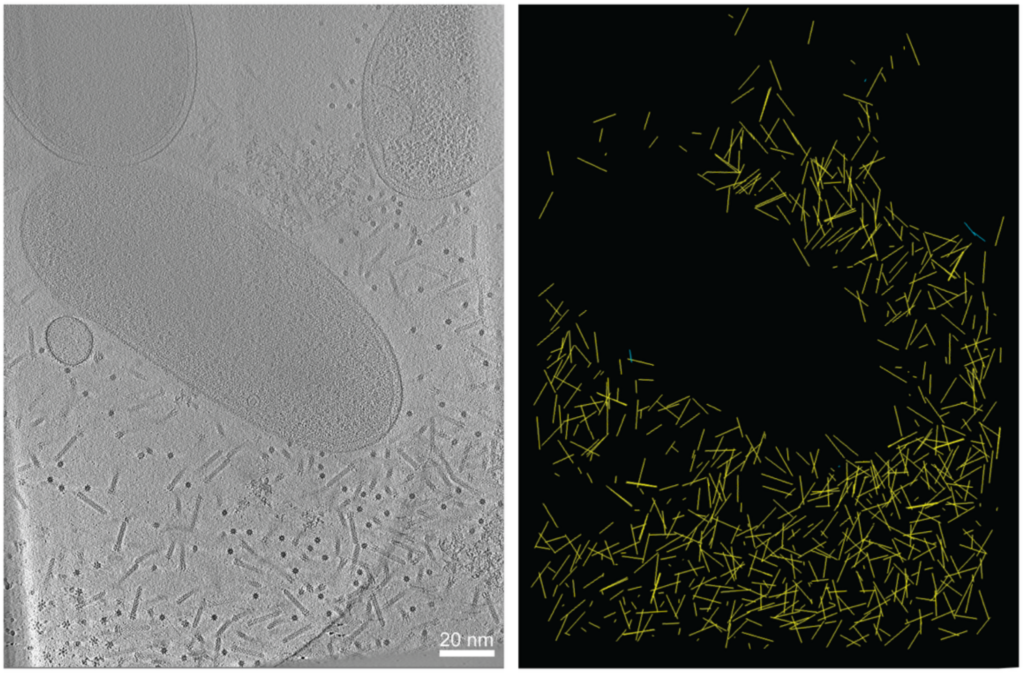

Figure R1. Cryo-tomogram of *E. pacifica* co-incubated with purified AlgoCIS.
The segmented pre- (yellow) and post-firing (blue) AlgoCIS are shown on the right. Note that the number of post-firing AlgoCIS is extremely low (0.6%, 5 out of 772 particles) in the purified AlgoCIS sample.

3. Line 94-95 and extended figure 4c: as there was only one replicate of SDS gel followed by mass spec analysis of one faint band, it may need proper label-free proteomic quantification with triplicate to conclude that there was less Alg16B post firing. Such quantification will also include the quantity of most other Alg proteins, which should remain the same and serve as controls. In addition, from the gel, there was a band missing in post-firing samples around size of 25 kDa, which could be interesting to know what it was.

As suggested, we repeated the experiment, followed by label-free proteomic quantification in triplicate. Mass spectrometry indicates that there was a (non-significant) 13% decrease in the amount of Alg16B when comparing the pre-firing sample to the post-firing AlgoCIS WT samples (Log_2 ratio of -0.20218, p-value = 0.202). During these experiments, we noticed that it is challenging to separate the free Alg16B from the preparation in samples of very small volumes. Therefore, these experiments were to some degree inconclusive, likely due to these technical challenges. We present these data below in Figure R2.

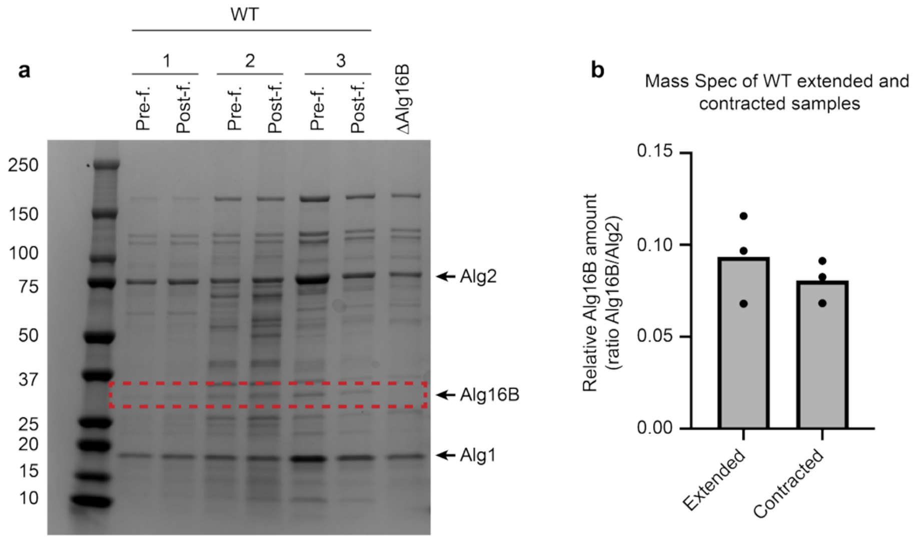

Figure R2. SDS-PAGE gel and mass spectrometry analysis of pre- and post-firing AlgoCIS. **a.** Replicates of the pre- and post-firing AlgoCIS samples on an SDS-PAGE gel. Note that the band around 25 kDa is present in all samples. **b.** Ratios of the iBAQ values from the label free proteomics that were normalized with the sheath value (Alg16B/Alg2).

We therefore decided to drop the original ED Fig. 3c, that showed the decreased amount of Alg16B as measured by mass spectrometry of an SDS gel band. That said, we believe that our structural data showing the absence of Alg16B in the fired confirmation strongly indicates the dissociation of Alg16B from the complex upon firing. We therefore suggest keeping this claim in the manuscript.

Finally, with regards to the lack of the 25 kDa band referred to by the reviewer, in our new replicates this band was always present (Figure R2).

4. State III (fully tilted tail fibers and closed cage): while there were quite a number of particles at state III (68%), however, when this state could be during the proposed stepwise process was not clear in Fig 5 or discussed enough. Especially compared to state II, where tail fibers were slightly tilted but cage has been already partially open. If state II was the stage before state III, then does

it mean that cage opens a bit then closes, and only when the tail fiber is fully tilted, then it reopens fully (entering state IV)?

The structure of state-II (slightly tilted tail-fibers and a partially open cage) indicates that the cage has some flexibility to slightly open, but needs the extra space from the tail-fiber tilting. We think that the cage can further close and only fully open upon binding to an as yet uncharacterized receptor.

To clarify this, we discussed more about the partially opening of the cage and the connection between state-II and state-III in the revised manuscript (line 207-213, 250-251).

5. Figure 5d: the state of 'fully tilted tail fibers, fully open cage, narrow iris' appeared to have close cage by the illustration above the text (if I understood correctly); also, what were the wavy lines indicating? Thus, it would be good to include the legend for color/shape representation of different proteins or structure parts in this figure.

We apologize for the confusion. The states in the bottom of figure describe the conformations after individual illustrated events. The cage in Fig. 5d will open after binding to an uncharacterized secondary receptor (wavy lines). To address this, we have revised the legends to make them clearer.

Other minor points:
1. Line 67 – 'suggesting a firing mechanism that may be distinct from pyocin R2': more explanation why authors hypothesize so will be appreciated for broader audience, since the checksum mechanism for pyocin R2 was also too briefly mentioned without more detailed comparison with the stepwise mechanism presented here.

We explained more detail about 'checksum' mechanism (line 45-46) and our rationale why there is a potential different firing mechanism of AlgoCIS (line 67-69) in the revised manuscript.

2. There was sometimes inconsistency in reference style. For example, line 138 (and several places throughout the text like line 233, 254 etc..) had style like 'pyocin R2 (ref.15)', which was different from the rest of the text.

Since there is a number before reference, many journals use an alternative reference style if the reference follows a number in the text.

3. Line 204: while AlgoCIS-Alg1DPM could not bind to bacterial cell surface, how about the non-contractile sheath mutant NC-AlgoCIS? If they bind, were there also different intermediate states observed?

We did observe the binding event when co-incubating AlgoCIS-Alg11^{DPM} with *E. pacifica*, but did not find any firing event (Please see ED Fig. 8c in the revised manuscript).

For NC-AlgoCIS, it is possible that we could discover different intermediate states when imaging *E. pacifica* co-incubated with the mutant. The performance of cryoET analysis, however, is insufficient to easily classify different intermediates as shown in our single particle analysis. Moreover, the characterization of different intermediates might need the higher resolution of

structures, which requires rigorous data leverages to discover additional intermediates using cryoET.

4. Line 350 – 'The position of the PAAR-like protein Alg10 is represented by dashed triangles': It should be clarified whether Alg10 was seen here by these structural analyses, or it was assumed that they were positioned there but not resolved within the analyses.

We have clarified in the revised version by stating "The position of the PAAR-like protein Alg10, which is not clearly observed in different intermediate states, is represented by dashed triangles."

5. Extended data figure 3a: noticeably, there were a large number of starfish shape-like arrangements of thin tubes, could authors indicate/speculate what they were within the figure?

The starfish-like structure is the dissociated baseplate complex. The attachment of baseplate to AlgoCIS is weak in the post-firing state, and its dissociation probably is caused by the compression of staining dye when performing negative staining. We added the related annotation in the figure legend.

Reviewer #3 (Remarks to the Author):
This paper tackles a central question in the field of type VI secretion systems and associated contractile injection systems: how is contraction/injection triggered and carried out? The authors choose Algoriphagus machipongonensis's AlgoCIS as a model system and start be determining the structure of the contracted form for comparison to a previously-published uncontracted form. They then investigate the role of the tail fibres, including an experiment to investigate whether tail fibre alone is sufficient to trigger contraction (it isn't). A series of serendipitous discoveries and rational insights subsequently lead the authors to capture a series of snapshots of the firing process of AlgoCIS: they design a non-contractile sheath mutant to stall the firing process and determined an intermediate structure using cryo-EM after firing was triggered. Negative staining of the same sample revealed two additional structural states. Finally, they generated a mutant lacking a disulphide bond in Alg11, which connects the cage and iris regions of the system, to investigate how the firing signal is transmitted through different CIS components. This led to the identification of another new structural state. By arranging these structures in sequence, the authors proposed a stepwise firing mechanism for the CIS.

The manuscript is overall very well-written, although perhaps a little lacking in details in places, likely an attempt to fit to word count. The science is good and I have no concerns, although some written commentary on choices would benefit the reader. Figures are overall very communicative although would benefit from additional information as described below.

We thank the reviewer for the positive feedback.

Major points

As mentioned repeatedly in my Minor points, I had some difficulty following ther narrative of the paper due to choices of what to put in Extended Data. In many cases I found that information essential to understanding the manuscript was in Extended, necessitating that flip back and forth to find it. I appreciate that length restrictions mean that some important data goes in Extended Data, but the core of a paper should be stand-alone and not absolutely require reference to essential data in Extended. Rearrangement of Figures is needed to render this manuscript more

easily accessible (the writing, on the other hand, is overall very good). In a similar vein in the text, many assumptions are not explained sufficiently for a non-expert reader understand the rationale behind choices, and a number of critical details are buried in Extended Data or Methods. I describe some, but not all, of these issues below.

As outlined below, we have revised some figures accordingly. In some cases, however, we prefer to keep the original versions, because we feel that otherwise the main figures become too complex, diluting the respective core message of the figure.

I also have concerns regarding the rationale for the interpretation of the stepwise order of firing. Given that the non-contractile sheath mutant stalls at state II, it seems that sheath contraction occurs prior to cage opening and tail fiber tilting, and is necessary for these conformational changes. Therefore, rather than reflecting the physiological firing pathway of the CIS in vivo, the transitions observed under negative staining may represent an alternative series of conformational changes occurring in the absence of sheath contraction. It would greatly clarify the proposed mechanism if the authors discussed the role of the sheath protein (Alg2) in mediating structural rearrangements during firing.

Previous studies on diverse CISs and phages (e.g. PMIDs: 15315755, 32350467) have shown that sheath contraction occurs after conformational changes of the baseplate. In such models, the conformational changes of the tail-fiber will induce the expansion of the baseplate iris, resulting in the expansion and rotation of sheath initiator protein. Since the sheath initiator has extensive interactions with the sheath protein, the conformational change of the sheath initiator will introduce a twist force onto the sheath, thereby initiating sheath contraction (line 119-121). The sheath contraction is the last and final step in the mechanism, propelling the inner tube to puncture targets. Our structures are fully consistent with such models. The reviewer argues that the step of sheath contraction could be upstream of and necessary for the induction of conformational changes in the baseplate/cage/tail fibers. If this was true, then we would not expect to observe states II/III/IV/V with the non-contractile mutant.

Similarly it isn't clear why the authors choose place so-called "stage III" after "stage II", given that the cage is more open in "stage II", which is arguably more important for injection of the tube that tail fibre pivoting.

In contrast to "stage-II," "stage-III" has the same conformation of the tail-fiber as the post-firing state (VI). This implies that the tail-fiber needs to fully tilt for firing. The partially open cage seen in "state-II' is likely due to the cage having some flexibility to slightly open when the tail-fibers tilt outwards slightly. We explain our rationale in the revised version (line 207-213).

Minor points

Introduction, line 44: the description of the "checksum" mechanism here fails to relate the name "checksum" to the described mechanism. For an audience who are not experts in the field, explanation of why the mechanism is referred to as "checksum" would be helpful.

See our response to Reviewer 2. We have revised accordingly to depict the detail of "checksum" mechanism.

Introduction, line 47: this paragraph uses the phrase “mode of action” to refer to the broader context in which they act. I’d argue a better phrase might be something like, “the context in which these CISs work differs”. To me, “Mode of action” strongly evokes their firing mechanism (the gap in knowledge that you seek to address).

The term “mode of action” has been used widely in the field of contractile injection system (PMIDs: 31597107, 39418385, 28818949, 35165386, 36894633, 31384001, et al.). We therefore think that it is important to keep the same term to make it clear for audience.

Results, line 73: Would be nice to have the nominal resolution stated instead of having to have readers search for this information.

See our response to Reviewer 1. We added the detail resolution in different parts of post-firing AlgoCIS.

Figure 1 (and Extended Data Figure 1): is the operon structure relevant? Consider removing? At the very least it should be a separate panel. I personally did not find that this contributed to the story, yet critical information was relegated to Extended Data figures (more on which below).

We think that it is essential to show the AlgoCIS gene cluster for context. Moreover, since the color code matches the one shown in the atomic model, we believe that it makes more easily accessible to a broad audience.

Results, line 81 / Figure 1: This is about comparison to the previously-published uncontracted state (indeed, the figure title is “AlgoCIS undergoes significant structural re-organization upon firing.”), but it feels awkward to flip between Extended Data Figure 1 and Figure 1 to make the comparisons between published and unpublished, and Figure 1 cannot stand alone. I’d strongly recommend combining the two figures into a larger figure, and include the electron density map for the unconstricted state. Extended Data should extend a figure, but not be an essential part of the figure that is relegated to the end of the paper: Extended Data Figure 1 is an essential part of Figure 1 and therefore deserves to be merged into the main figure.

Thanks for this suggestion. We have incorporated this into current Fig. 1.

Line 111 and throughout: As a non expert, I struggled to understand what structures correspond to the “cage” - please highlight in Figure 1. An interpretative cartoon would be invaluable here (like the last column of Fig. 4), and include definition of the iris (which is currently defined in Fig. 2).

We revised Fig. 1 to highlight the “cage” and “iris” and included a cartoon of the post-firing AlgoCIS accordingly.

Figure 2: my comments are the same here as Figure 1. Figure 2 currently does not stand alone, and needs Extended Data Figure 4 to be merged into it. A detail panel of Alg19 pivoting might also be helpful here instead of referring back to a part of Fig. 1?

ED Fig. 3 (in the revised manuscript) mainly shows that cap adaptor protein Alg16B is absent in the post-firing AlgoCIS, which does not fit to the tail-fiber part shown in Fig. 2.

To avoid referring back to Fig.1, we added a new panel (new ED Fig. 5b) to compare the different conformations of one tail-fiber in pre- and post-firing AlgoCIS.

Line 163: I would be interested to hear your speculations on why any AlgoCIS can bind without Alg19?

For AlgoCISΔAlg19, we often observed particles lying on the bacterial surface instead of being oriented perpendicularly. Thus, it suggests that the sheath might interact with bacteria non-specifically (Figure R3a). To explore this, we analyzed the Alphafold predicted structure of sheath protein Alg2 and found that the domain IV of Alg2, which is exposed on the sheath surface, is negatively charged (Figure R3b). We therefore speculate that AlgoCIS could interact with bacteria non-specifically via the sheath.

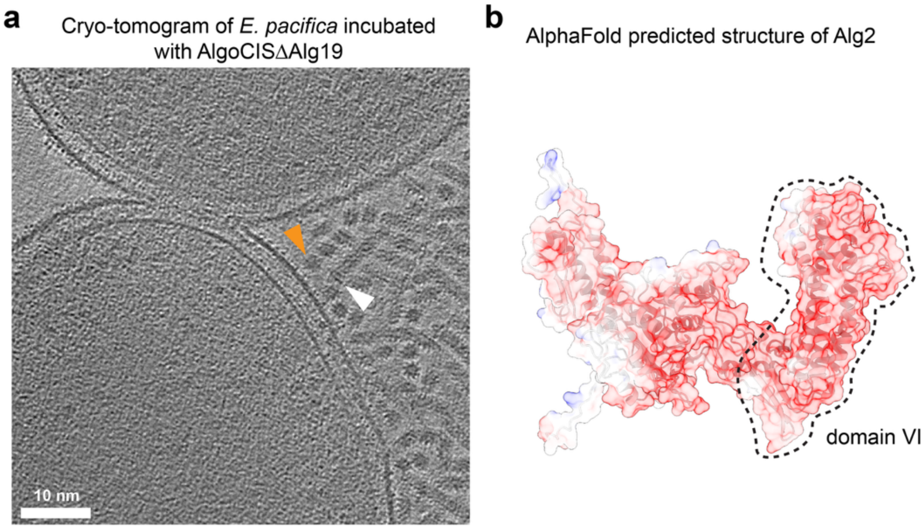

Figure R3. AlgoCIS can non-speciafically interact with bacterial surface via the sheath.
a. Cryo-tomogram of *E. pacifica* co-incubated with purified AlgoCISΔAlg19. Several particles (orange arrowhead) were observed to lie on the bacterial surface. The tail-fiber less baseplate is indicated by white arrowhead. **b.** Shadowed surface with electrostatic potential analysis (blue: positively charged; red: negatively charged) of the AlphaFold predicted structure Alg2, showing that the domain VI (dashed box) exposed outside is negatively charged.

Line 170: Nice experiment!

Thank you.

Line 182: I'm not sure if "generating" insights is quite the right phrase? "Attain" insights, or "generate data"?

We have revised accordingly.

Figure 4: Figure 4 feels more like an interpretative figure than a data figure. Again, this would benefit from incorporating some of Extended Data Figure 9. Perhaps the more interpretative aspects could be moved to Figure 5, which is already semi-redundant with Figure 4, and Figure 4 fleshed-out with more actual data?

ED Fig. 8 (in the revised manuscript) mainly analyzed the iris structures in state-III to state-V. Given several states shown in Fig. 4, incorporation of some data in ED Fig. 8 will make the figure more complex and audience might not easily be able to follow. We therefore would like to keep the original version.

Line 193: I agree that this is exciting, and appreciate that some peculiarity of the system necessitates negative stain work. I would appreciate a little more interpretative context from the authors, though: why do you think negative stain was required for these insights? Do you have concerns over whether these may be artefacts? If not, why not? I don't view these as issues, but think the paper would be improved with this commentary.

We have screened different conditions for cryo-sample preparation but failed to obtain these two states (state-III and state-IV) seen in negative-stain EM. We instead determined the state-II, where tail-fibers are slightly outward-tilted. Given this observation and that tail-fibers fully tilt in states-III and -IV, we hypothesize that the particles in these two states were possibly blotted away by filter papers when preparing cryo-samples.

To clarify this, we added our speculation in the revised text (line 203-205).

Line 201: I'd have appreciated some context. Why did you think this disulphide bond is relevant to firing?

To elucidate how the signal of cage opening propagates to the iris, we first carefully checked binding profiles between baseplate iris and cage. We unexpectedly found the disulfide bond, which is generally stable. We therefore further explored this and found that it is essential in the signal transmission from the cage to the iris.

Line 203: Define "DPM".

DPM: double point mutant. We added the explanation accordingly.

Fig. 5C: How do the tail fibres first bind to the outer leaflet of the outer membrane – which is NOT fluid – and subsequently splay out to enable the cage to come to close proximity with the membrane? Once bound the tail fibres would be locked immobile by LPS.

Given that tail-fibers have IgG-rich domain, we think that tail-fibers can reversibly interact with the bacterial outer membrane. AlgoCIS therefore may be able to use its tail-fiber to walk/re-orient on the bacterial surface in a similar manner as T4 phage (PMID: 26283379). When several tail-fibers attach to bacterial surface, the structural flexibility of tail-fibers possibly brings the baseplate close to bacterial surface.

Reviewers’ comments in black
Authors’ responses in blue

Reviewer #2 (Remarks to the Author):
The work by Xu et al used high-resolution cryo-EM with structure-guided engineering to reveal stepwise mechanisms of the firing and contraction of a bacterial contractile injection system, AlgoCIS. The contraction of CISs was previously poorly understood, however, this manuscript has revealed a novel mechanism, which is distinct from the previous 'checksum' mechanism proposed for pyocin R2.

With the new revision, authors have successfully addressed all my review comments and confusions I had (I really appreciate their clear explanation in addressing comments, not only in the manuscript, but also in the letter). With the revision, I do not have further major comments (with only one minor comment listed below). In general, with such extensive solid data on the high resolution structural analyses, the work is very impressive and convincing. This study significantly expands and deepens our current understanding on the firing of CISs and will guide future investigations into further mechanical insights of the contraction of other CISs. Moreover, I also believe that it will be of interest to a wide audience. For example, this cleverly designed structure-guided engineering approach could inspire future efforts to “lock” other dynamic nanomachines at specific stages, enabling the resolution of stepwise mechanisms that are otherwise difficult to capture due to their too dynamic, transient or subtle conformational changes.

minor comment: since the author decides to remove the original ED fig. 4c of the SDS PAGE and Mass spec analysis on the pull-down assays, perhaps majority of the method section on this part can be removed (line 654-672), unless it is relevant to other data.

We appreciated the positive feedback from the reviewer and have removed the part of mass spectrometry analysis from the method section accordingly.

Reviewer #3 (Remarks to the Author):
I am happy with the author's revisions. Very nice!

We thank the reviewer for constructive feedback during peer-review.
